# Munc13- and SNAP25-dependent molecular bridges play a key role in synaptic vesicle priming

**DOI:** 10.1126/sciadv.adf6222

**Published:** 2023-06-21

**Authors:** Christos Papantoniou, Ulrike Laugks, Julia Betzin, Cristina Capitanio, José Javier Ferrero, José Sánchez-Prieto, Susanne Schoch, Nils Brose, Wolfgang Baumeister, Benjamin H. Cooper, Cordelia Imig, Vladan Lučić

**Affiliations:** ^1^ Department of Molecular Structural Biology, Max Planck Institute of Biochemistry, 82152 Martinsried, Germany.; ^2^ Department of Neuropathology, University Hospital of Bonn, 53127 Bonn, Germany.; ^3^ Departamento de Bioquímica y Biología Molecular, Facultad de Veterinaria, Universidad Complutense, and Instituto de Investigación Sanitaria del Hospital Clínico San Carlos, 28040 Madrid, Spain.; ^4^ Department of Molecular Neurobiology, Max Planck Institute of Multidisciplinary Sciences, City Campus, 37075 Göttingen, Germany.; ^5^ Department of Neuroscience, University of Copenhagen, 2200 Copenhagen, Denmark.

## Abstract

Synaptic vesicle tethering, priming, and neurotransmitter release require a coordinated action of multiple protein complexes. While physiological experiments, interaction data, and structural studies of purified systems were essential for our understanding of the function of the individual complexes involved, they cannot resolve how the actions of individual complexes integrate. We used cryo–electron tomography to simultaneously image multiple presynaptic protein complexes and lipids at molecular resolution in their native composition, conformation, and environment. Our detailed morphological characterization suggests that sequential synaptic vesicle states precede neurotransmitter release, where Munc13-comprising bridges localize vesicles <10 nanometers and soluble *N*-ethylmaleimide–sensitive factor attachment protein 25–comprising bridges <5 nanometers from the plasma membrane, the latter constituting a molecularly primed state. Munc13 activation supports the transition to the primed state via vesicle bridges to plasma membrane (tethers), while protein kinase C promotes the same transition by reducing vesicle interlinking. These findings exemplify a cellular function performed by an extended assembly comprising multiple molecularly diverse complexes.

## Teaser

Single nanometer in situ imaging shows that synaptic vesicle tethers of different composition organize steps towards fusion.

## INTRODUCTION

Similar to most other cellular processes, synaptic vesicle (SV) tethering, priming, and fusion require a precise orchestration of molecular pathways carried out by multiple macromolecular complexes organized in functional units. The last step of the neurotransmitter release process, SV fusion with the plasma membrane, most often occurs at a specialized region of the presynaptic terminal apposed to the synaptic cleft, termed the active zone (AZ). Before fusion, SVs are recruited to and held in close proximity to the AZ membrane, involving the tight coupling of the neurotransmitter release machinery, SVs, and voltage-gated calcium channels (*1*–*5*).

The molecular mechanisms operating at AZs have long been a focus of nerve cell biology. Here, the Munc13-1 and Munc13-2 members of the Munc13 protein family are necessary for SV priming, i.e., for the formation of a readily releasable pool of SVs, a physiologically defined process that renders individual SVs capable of fusion upon Ca^2+^ influx into the presynaptic terminal (*6*, *7*). The RIM protein family has a central role in the organization of the AZ (*2*, *8*). RIM1α reverses Munc13-1 homodimerization, thus activating Munc13-1 (*9*). Munc13s and Munc18-1 facilitate the formation of the synaptic soluble *N*-ethylmaleimide–sensitive factor attachment protein (SNAP) receptor (SNARE) complex comprising SNAP25, syntaxin-1, and synaptobrevin-2 (*10*). The ultimate fusion of primed SVs is driven by the full assembly of the SNARE complex (*3*, *11*). Munc13-1 is thought to execute its SNARE-regulating priming function via its MUN domain and is stimulated by Ca^2+^-calmodulin binding to an amphipathic helical motif, by diacylglycerol (DAG) binding to a C_1_ domain, and by Ca^2+^-phospholipid binding via the central C_2_B domain (*12*–*17*). Reconstitution assays showed that efficient liposome fusion requires C_1_, C_2_B, MUN, and C_2_C domains of Munc13-1, leading to the notion that Munc13-1 can simultaneously bind SVs, plasma membranes, and SNAREs (*4*).

Our current understanding of presynaptic AZ function is based on comprehensive cell biological and functional studies, typically involving the combination of targeted genetic perturbations with morphological, ultrastructural, and electrophysiological analyses. However, the mechanism by which individual steps of the transmitter release process are coordinated in the synapse, the identity, and positioning of relevant protein machines remains the subject of speculation (*4*). One major knowledge gap is how the molecular SV tethering, priming, and fusion machinery are organized to drive activity-dependent membrane trafficking and neurotransmitter release. Addressing this issue experimentally in a cellular context is nontrivial because it necessitates visualizing both lipids and protein complexes at molecular resolution in situ within the complex environment of the presynaptic AZ.

Cryo–electron tomography (cryo-ET) provides a molecular-level visualization of the presynaptic terminal (*18*) and is uniquely suited for simultaneous, label-free imaging of unstained molecular complexes at single-nanometer resolution in situ, i.e., in their native environment (*19*, *20*). Previous studies showed that (i) pleomorphic protein bridges organize SVs by interlinking (via molecular connectors) and tethering them (via molecular tethers) to the presynaptic plasma membrane, (ii) the distance between SVs and the AZ membrane and the tethering state of the SVs are indicative of SV progression toward fusion (*21*, *22*), and (iii) tethers aid in localizing neurotransmitter release sites near postsynaptic receptors (*23*).

To avoid the present nomenclature ambiguity, here, we define tethers in the structural sense, as all directly observed bridges linking SVs with the AZ membrane (*21*, *24*). The term tether was used to describe an array of different biological structures. In the secretory system, tethers were defined functionally, without being observed, as factors upstream of the SNARE complex formation. This terminology poses problems in cryo-ET because molecular identity cannot be assumed a priori. In addition, we refrain from using “docking” because in cryo-ET, SVs are not seen making direct, extended contact with the plasma membrane (*18*).

The vast majority of our insights into synaptic ultrastructure stems from transmission electron microscopy (EM) studies of dehydrated and plastic-embedded synapses. Multiple steps in the preparation of such samples (i.e., chemical fixation, dehydration, heavy metal staining, plastic embedding, and mechanical sectioning) can introduce structural artifacts that alter or conceal ultrafine molecular arrangements in the synapse. Quick freezing and deep-etching preparations avoid chemical fixation providing better preservation, which resulted in the first observation of filaments interconnecting and tethering SVs and led to the proposal that these filaments may constrain the movement and cluster SVs (*25*–*27*). The vitrification by high-pressure freezing followed by freeze substitution (HPF/FS) further limited the extent to which preparation artifacts become manifest (*28*). Because this method still precludes molecular interpretation, ultrastructural analyses of pre-SV pools in mutant synapses have focused predominantly on measuring distances between vesicles and the plasma membrane or on the distribution of long, filamentous structures that are detectable with heavy metal–based staining protocols (*29*–*33*). Specifically, deletion of Munc13 or individual SNARE proteins in synapses alters the distribution of vesicles at the AZ and reduces the number of vesicles making an apparent physical contact with the AZ plasma membrane (*30*, *31*, *34*, *35*).

Here, we performed structural molecular imaging of priming-deficient synapses by cryo-ET to resolve the mechanism for organizing AZ-proximal SVs, determine the vesicle tethering functions of Munc13s, SNAP25, and RIMs, and identify some of the tethers. On the basis of our findings, we propose a new model of the molecular mechanisms mediating the SV progression toward fusion, where RIMs, Munc13s, and SNAREs are required in subsequent steps that lead from SV tethering, via priming, to fusion.

## RESULTS

### Munc13 and SNAP25 do not affect the overall distribution of SVs

Deletion of Munc13 and SNAP25 proteins in neurons leads to complete abolition and severe impairment of release, respectively, and a loss of fusion-competent vesicles (*7*, *36*, *37*), as well as a redistribution of SVs in the vicinity of the AZ plasma membrane (*30*, *31*, *35*).

Most of the previous synaptic cryo-ET studies were performed on synaptosomes, a well-established model that responds to pre- and postsynaptic stimulation (*23*, *38*–*41*), preserves the macromolecular architecture, and is an excellent substrate for cryo-ET imaging at the molecular level (*18*, *42*–*44*). Because Munc13 and SNAP25 knockout mice die at birth, we prepared embryonic hippocampal organotypic slices and cultured them for 3 to 5 weeks before synaptosome preparation. We detected and imaged synaptosomes directly in the crude synaptosomal fractions. These could not be visually distinguished from gradient centrifugation–based synaptosomes, with the added advantage that avoiding the gradient centrifugation step shortened the preparation time as compared to our previous studies (*22*).

**Fig. 1.**
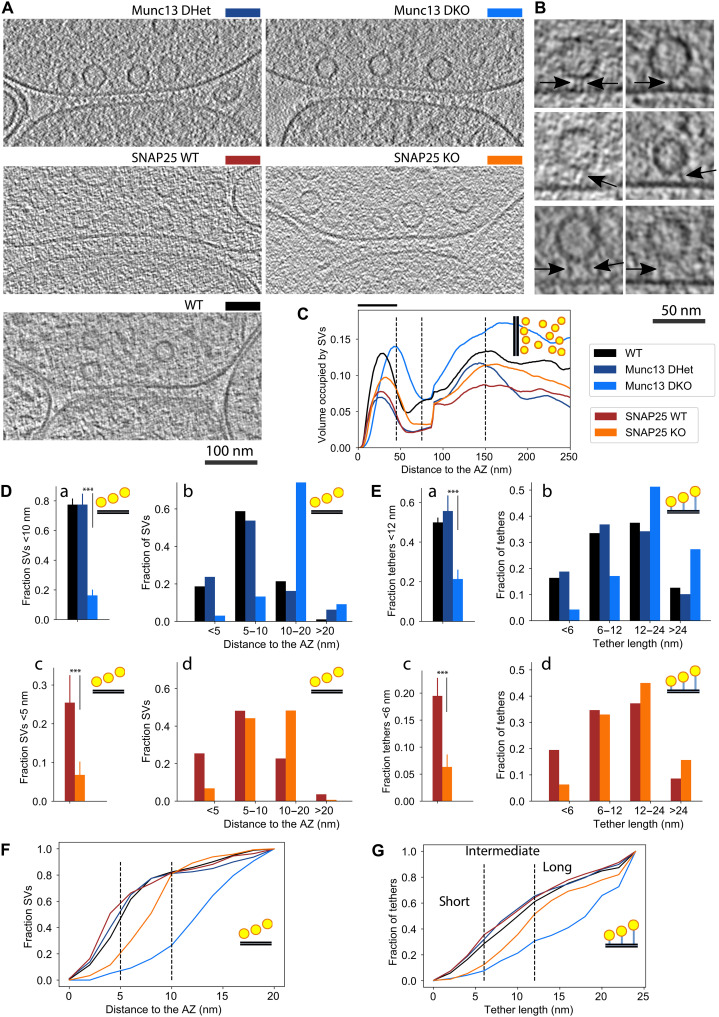
Munc13 and SNAP25 influence the proximal SV location and tether length. (**A**) Cryo-ET slices of synapses from different conditions, as indicated by the legend. (**B**) Magnified cryo-ET slices showing examples of proximal SVs and tethers: SVs located <5 nm (short tethers) (top), 5 to 10 nm (intermediate tethers) (middle), and further than 10 nm to the AZ membrane (long tethers) (bottom). The images are from WT (top and middle) and Munc13 DKO synapses (bottom). Arrows point to tethers. (**C**) SV distribution up to 250 nm to the AZ membrane. The horizontal bar above the graph indicates the location of the proximal zone. (**D**) Fraction of proximal SVs <10 nm (a) and 5 nm (c) to the AZ membrane and histograms of the proximal SV distances where bins represent SV distance classes (b and d). (**E**) Fraction of tethers shorter than 12 nm (a) and 6 nm (c) to the AZ membrane and histograms of tether lengths where bins represent short, intermediate, and long tethers (b and d). (**F**) Cumulative distribution function of the SV distances, where the distances used to classify the proximal SV distances are shown by black, dashed lines. The data shown here are the same as the data shown in histograms in (C). (**G**) Cumulative distribution function of tether lengths, where the lengths used to classify tethers are shown by black, dashed lines. The data shown here are the same as the data shown in histograms in (E). ***P < 0.001

Cryo–electron tomograms of synaptosomes showed smooth and continuous membranes, spherical SVs at a distance to the plasma membrane, a well-defined synaptic cleft, and no signs of cytoplasmic aggregation (Fig. 1A), as expected from vitrified samples and in accordance with previous studies (movie S1) (*18*). Each presynaptic terminal contained many SVs, a mitochondrion, and an apposed postsynaptic terminal containing a postsynaptic density characteristic of excitatory synapses.

We compared synapses from mice lacking both dominant Munc13 isoforms (Munc13-1 and Munc13-2) (Munc13 DKO) (movie S2) and Munc13 double heterozygous (DHet) littermate controls. As this breeding strategy did not enable us to obtain wild-type (WT) littermate controls, we prepared and processed postnatal day 0 (P0) organotypic slices from C57BL6/N mice in parallel (WT). We further imaged hippocampal organotypic slice synaptosomes from mice lacking SNAP25 (SNAP25 KO) (*37*) and their littermate controls (SNAP25 WT).

Under all conditions imaged, SV concentration profiles showed an accumulation of SVs in the proximal zone (45 nm from the AZ membrane), a decreased concentration in the intermediate zone (45 to 75 nm to the AZ membrane), and again an increased concentration in the distal zone (75 to 250 nm to the AZ zone) (Fig. 1C). We previously observed that a perturbation of this pattern indicates a massively disturbed SV organization and neurotransmitter release (*21*, *22*). Furthermore, the surface concentration of proximal SVs of synapses lacking Munc13 and SNAP25 was very similar to their controls (Munc13 DHet and SNAP25 WT, respectively) (fig. S1A). The higher concentration observed under the WT condition in comparison to other control conditions was likely caused by the different genetic background or the different age of the animal on the day of culturing [embryonic day 18 (E18) versus P0]. In sum, genetic ablation of Munc13 and SNAP25 neither changed the overall organization of SVs, here defined as the shape of the SV concentration at a precision of >20 nm, nor their concentration at the proximal zone.

### Munc13 and SNAP25 organize AZ-proximal SVs

We then investigated whether Munc13 and SNAP25 influence the SV organization within the proximal zone. The distance between proximal SVs and the AZ membrane was 14.3 ± 0.4 nm (means ± SEM) in Munc13 DKO synapses, significantly larger than in Munc13 DHet (8.4 ± 0.6 nm; *P* < 0.001, *t* test) and WT (8.3 ± 0.3 nm) synapses (fig. S1Ba).

Probability distributions (normalized histograms) of the SV distances showed peaks located around 16 nm for Munc13 DKO and around 6 nm for Munc13 DHet synapses, indicating that there are at least two AZ distance-dependent SV states (Fig. 1Db and fig. S1Bc). The optimal histogram peaks separation distance, also determined as the most pronounced difference between the corresponding cumulative distribution functions, was at ∼10 nm (Fig. 1F).

To confirm that the distance of 10 nm provides a valid separation of the two states, we calculated the fraction of proximal SVs in the two states. Fifteen percent of proximal SVs in Munc13 DKO synapses were within <10 nm of the AZ membrane, a 4.7-fold decrease from Munc13 DHet and WT synapses (80% SVs within 10 nm) (*P* < 0.001, χ^2^ test) (Fig. 1Da). In line with this finding, the fraction of proximal SVs closer than 5 nm was 7.6 times larger in Munc13 DHet than in Munc13 DKO synapses (*P* < 0.001, χ^2^ test) (fig. S1Bb). Together, our data indicate that Munc13 deletion leads to a shift in the distribution of SVs of approximately 10 nm away from the AZ.

We found that the removal of SNAP25 increases the mean proximal SV distance to the AZ (from 8.2 ± 0.5 nm in SNAP25 WT to 9.8 ± 0.3 nm in SNAP25 KO; *P* = 0.002, *t* test) (fig. S1Ca). As for the Munc13 conditions, histograms showed distinct peaks (Fig. 1Dd and fig. S1Cc), but the most pronounced difference was found at approximately 5 nm, confirmed by the cumulative SV distance distributions (Fig. 1F). Consistent with these results, the fraction of SVs located <5 nm to the AZ membrane was significantly reduced in the absence of SNAP25 (3.7 times smaller, from 25% in SNAP25 WT to 7% in SNAP25 KO synapses; *P* < 0.001, χ^2^ test) (Fig. 1Dc), while the fraction of SVs <10 nm to the AZ membrane was 1.4 times smaller in SNAP25 KO synapses (*P* < 0.001, χ^2^ test) (fig. S1Cb).

The determined optimal SV distance peak separation values among WT, SNAP25, and Munc13 synapses (5 and 10 nm) have an inherent uncertainty because the fraction of SVs closer to the AZ calculated for similar values (e.g., 4 and 9 nm) was also significantly different (*P* < 0.001, χ^2^ test for both SNAP25 KO and Munc13 KO synapses, respectively) (fig. S1, Bc and Cc). The inflection points of the cumulative distributions (Fig. 1F), determined as the middle points of the regions of steep increase, were located at 3 nm for WT, 8 nm for SNAP25 KO, and 13 nm for Munc13 DKO synapses and may be interpreted as characteristic SV distances for these conditions.

Together, we found that the removal of Munc13 and SNAP25 increased the SV distance to the AZ with a single-nanometer precision (Fig. 1, Db, Dd, and F); that is, WT synapses are characterized by SV distances of 0 to 5 nm, SNAP25 KO by 5 to 10 nm, and Munc13 DKO by >10 nm. In addition, both the spatial extent and the magnitude of the SV depletion in the proximal zone caused by Munc13 deletion were greater than those after SNAP25 removal.

### Munc13 and SNAP25 control the SV tether types

Next, we tested whether Munc13 and SNAP25 affect tether morphology. Visual inspection confirmed our previous observations that SV tethers were the main structural elements organizing SVs at the AZ membrane (Fig. 1B) (*18*, *21*). SV tethers were computationally detected in an automated, unbiased manner using hierarchical connectivity segmentation, as clusters of connected pixels that link two membranes in three-dimensional (3D) at multiple thresholds (*45*). Tether length was measured between the closest protein-membrane contacts, taking tether curvature into account. We found that tether length was significantly increased in Munc13 DKO (20.9 ± 1.2 nm, means ± SEM) compared to Munc13 DHet (13.1 ± 0.5 nm) and SNAP25 KO (15.7 ± 0.5 nm) compared to SNAP25 WT (12.7 ± 0.4 nm) synapses (*P* < 0.001 in both cases, *t* test) (fig. S1, Da and Ea).

The cumulative distribution of tether lengths showed a clear separation at approximately 6 and 12 nm (Fig. 1G), which is 20% larger than the previously defined SV distance limits (5, 10, and 20 nm). Consequently, we classified tethers by their length into <6 nm (short tethers), 6 to 12 nm (intermediate), and 12 to 24 nm (long) groups. Using this classification, we found that the fraction of all tethers shorter than 12 nm was significantly decreased in Munc13 DKO synapses, as was the fraction of all tethers shorter than 6 nm in SNAP25 KO synapses (*P* < 0.001 in both cases, *t* test) (Fig. 1, Ea and Ec), which can be also seen on tether length histograms (Fig. 1, Eb and Ed, and fig. S1, Dc and Ec). We also obtained very significant results for tether lengths of 5 nm (SNAP25) and 10 nm (Munc13) (both *P* < 0.001 *t* test).

Short tethers remaining in SNAP25 KO and Munc13 DKO synapses could not be visually distinguished from those present under other conditions. However, their low number precludes accessing whether these two groups represent different tether species.

In sum, our SV tether analysis showed that deletion of Munc13 or SNAP25 strongly and differentially increases tether length, that Munc13 deletion caused stronger effects, and that these changes parallel alterations in AZ-proximal SV localization. Therefore, we defined three Munc13- and SNAP25-dependent SV tethering and localization states. Namely, SVs localized <5 nm to the AZ membrane and characterized by short tethers belong to the SNAP25-dependent state, SVs localized >10 nm to the AZ membrane and characterized by long tethers belong to the Munc13-independent state, and the SVs localized 5 to 10 nm to the AZ membrane with intermediate tethers belong to the intermediate state.

### The lack of all major RIM family proteins severely impairs SV organization

Deletion of RIM proteins, the core components of the presynaptic AZ, results in a severe impairment of release and a strong reduction in the number of SVs in the vicinity of the AZ zone membrane, as assessed by EM of chemically fixed samples (*46*). We examined the role of RIMs (*8*) in SV organization at the AZ using cryo-ET of synapses lacking all four multidomain RIM isoforms, 1α, 1β, 2α, and 2β. Because mice deficient for all RIM1 and RIM2 variants die after birth (*46*), we prepared synaptosomes from cultured primary neurons of double-floxed mice (RIM1/2^flox/flox^) and compared synapses from neurons expressing active (RIM cDKO) or inactive (RIM Ctrl) Cre recombinase.

The mean SV distribution profiles in RIM Ctrl synapses showed the expected features, i.e., an accumulation of SVs in the proximal zone and a lower SV concentration in the intermediate zone, while both of these features were lacking in RIM cDKO samples, in agreement with the visual assessment (fig. S2, A and B). Inspection of SV profiles in individual synapses showed that none of the five RIM cDKO synapses had these features, contrary to all four RIM Ctrl synapses assessed. Although two of four RIM Ctrl synapses were visually assessed to be inhibitory, their SV distribution profiles were indistinguishable among themselves and in comparison to other excitatory WT synapses (Fig. 1C). Together with the observations that all five RIM cDKO synapses were excitatory and that their SV distribution profiles were all very different from the WT, our data argue that the differences between RIM Ctrl and RIM cDKO synapses are not a consequence of a different neurotransmitter type.

The distribution of the proximal SV distances to the AZ membrane was shifted toward higher distances in RIM cDKO synapses as compared to Ctrl synaptosomes (*P* = 0.0064, χ^2^ test) (fig. S2C). The fraction of SV <10 nm to the AZ membrane was significantly smaller in RIM cDKO tomograms (*P* = 0.0036, *t* test). The surface concentration of proximal SVs at the AZ membrane was 2.4 times smaller in RIM cDKO synapses, but this difference did not reach significance because of the high variance (*P* = 0.18, *t* test).

Our data indicate that the overall synaptic distribution of SVs and their concentration in the proximal zone is severely disturbed in synapses lacking all RIM1 and RIM2 isoforms, even more so than what we had observed previously in RIM1α KO synapses (*22*). Furthermore, as the cumulative distribution of SV distances in RIM cDKO synapses was rather flat up to 80 nm to the AZ membrane (fig. S2D), it did not show an inflection point, suggesting a lack of structures that could constrain SV distance to the membrane in RIM cDKO synapses.

### 4-β-Phorbol-12,13-dibutyrate supports the SNAP25-dependent state

Phorbol esters, including 4-β-phorbol-12,13-dibutyrate (PDBu), are analogs of the endogenous second messenger DAG and known to bind and activate Munc13 and other C_1_ domain proteins, leading to a potentiation of neurotransmitter release in a Munc13-dependent manner (*12*, *47*, *48*). To elucidate structural correlates of this potentiation, we analyzed cryo-ET images of untreated and PDBu-treated neocortical synaptosomes from adult WT mice. Furthermore, we also examined synapses treated with PDBu together with either protein kinase C (PKC) inhibitor Ro31-8220, or calphostin C, an inhibitor of DAG/PDBu binding to C_1_ domains of Munc13 and PKC.

Analyses of neocortical synaptosomes in the absence of stimulation confirmed that PDBu increases glutamate release. This effect was prevented by calphostin C, but not by specifically inhibiting PKCs using Ro31-8220 and BID1 (fig. S3A), as shown previously (*49*). Furthermore, glutamate release from PDBu-treated synaptosomes stimulated by 5 mM KCl was increased upon the addition of extracellular Ca^2+^ (fig. S3B).

**Fig. 2.**
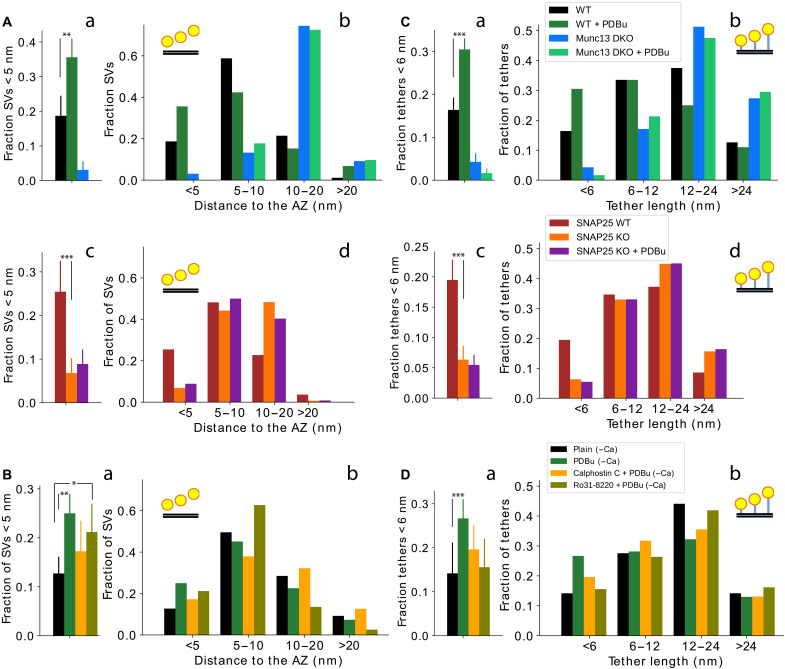
PDBu influences the proximal SV localization and tether length. (**A**) Fraction of proximal SVs located <5 nm to the AZ membrane (a) and histograms of SV localizations for Munc13 and SNAP25 conditions (b). (**B**) The same as (A), but for pharmacologically treated neocortical synaptosomes. (**C**) Fraction of short tethers (<6 nm) (a) and tether length histograms (b) for Munc13 and SNAP25 conditions. (**D**) The same as (C), but for pharmacologically treated neocortical synaptosomes. *P < 0.05; **P < 0.01; ***P < 0.001

We then tested whether application of PDBu influences SV tethering as assessed by cryo-ET. To prevent spontaneous vesicle fusion after PDBu potentiation, we subjected synaptosomes to an extracellular Ca^2+^-free solution. We found that neither PDBu alone nor PDBu in combination with calphostin C or Ro31-8220 affected the overall SV distribution or the surface concentration of proximal SVs (fig. S4A). However, the proximal SV distance to the AZ membrane was significantly decreased by PDBu (*P* = 0.037, *t* test) (fig. S4B) caused by the significantly higher fraction of proximal SVs of PDBu-treated synapses located <5 nm to the AZ membrane (*P* = 0.0017, χ^2^ test) (Fig. 2, Ba and Bb). The lack of significant changes upon combined calphostin C + PDBu treatment indicates that the PDBu-induced SV translocation we observed is mediated by Munc13 C_1_ domain activation.

Furthermore, the proximal SV distance to the AZ membrane was strongly decreased by combined Ro31-8220 + PDBu as compared to control values (*P* < 0.001, *t* test), even more so than by PDBu alone (fig. S4B). This change was evident for SV distances up to 10 nm from the AZ membrane, as can be seen from the histogram of proximal SV distances (Fig. 2Bb) and from the data showing that the fraction of Ro31-8220 + PDBu proximal SV is significantly increased for SVs <5 nm (Fig. 2Ba) and very significantly increased for SVs <10 nm to the AZ membrane (*P* = 0.040 and *P* < 0.001, respectively, χ^2^ test) (fig. S4B). Together, these observations indicate that PKC primarily affects SVs within 5 to 10 nm from the AZ membrane.

Tether length was significantly decreased by PDBu (*P* = 0.013, *t* test) (fig. S4C). This decrease was most prominent for short tethers (*P* < 0.001, χ^2^ test) (Fig. 1, Da and Db) and less so but still significant for tethers up to 12 nm in length (*P* = 0.003, χ^2^ test) (fig. S4C). Neither calphostin C + PDBu nor Ro31-8220 + PDBu resulted in significant differences. The latter is unexpected because Ro31-8220 + PDBu significantly decreased SV distance to the AZ membrane (Fig. 2Ba and fig. S4B) and PDBu alone significantly decreased tether length (Fig. 2Da and fig. S4C).

In sum, we found that PDBu activation of Munc13 reduces the proximal SV distance to the AZ and the tether length in a way that supports the SNAP25-dependent SV tethering state. Furthermore, our data indicate that PKC may contribute to SV localization by a tether-independent mechanism.

### PDBu-mediated regulation of SV localization and tether length is Munc13 and SNAP25 dependent

To clarify the molecular mechanism of the PDBu effects on SV localization and tethers, we analyzed Munc13 and SNAP25 deficient synapses after PDBu treatment. As expected, the application of PDBu on WT synaptosomes caused a significant increase in fractions of proximal SVs <5 nm to the AZ membrane (*P* = 0.007, *t* test) and short tethers (*P* = 0.005, *t* test) (Fig. 2, Aa, Ab, Ca, and Cb, and fig. S4I). Accordingly, the probability and the cumulative distribution of SV distances shifted toward smaller values (fig. S4F). These data are very similar to those presented in the previous section (Fig. 2, B and D) although different extracellular Ca^2+^ concentrations were used (i.e., 1.2 mM Ca^2+^ here versus nominally Ca^2+^-free in previous section).

In contrast, we found that neither the localization of SVs within the proximal zone nor their tether lengths were changed in PDBu-treated Munc13 DKO and SNAP25 KO synapses (Fig. 2, A and C, and fig. S4, D and E). There were no changes for any of the SV distance and tether length bins nor for the corresponding cumulative distributions (fig. S4, F to H). Therefore, these data confirm the observation that PDBu supports the SNAP25-dependent SV tethering state and shows that this effect requires both Munc13 and SNAP25.

### Munc13, SNAP25, and PDBu increase SV tethering

We previously showed that the increase in the number of tethers per SV is indicative of the SV progression toward fusion (*21*). Therefore, we tested whether our genetic and pharmacological treatments influence this relationship.

**Fig. 3.**
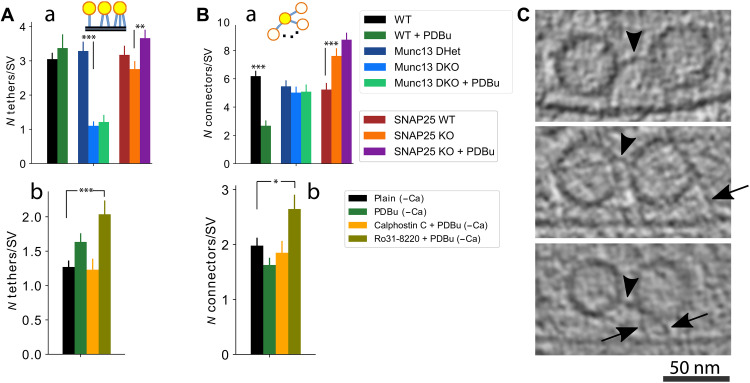
Munc13, SNAP2, PDBu, and PKC inhibition influence the number of SV tethers and connectors. (**A**) Number of tethers per proximal SV for Munc13 and SNAP25 conditions (a) and pharmacologically treated neocortical synaptosomes (b). (**B**) Number of connectors per proximal SVs for the same conditions as in (A). (**C**) Cryo-ET slices showing examples of SV connectors. Arrows point to tethers and arrowheads point to connectors. *P < 0.05; **P < 0.01; ***P < 0.001

We found that the number of tethers per proximal SV was reduced from 3.3 ± 0.3 in Munc13 DHet to 1.1 ± 0.1 (means ± SEM) in Munc13 DKO synapses (*P* < 0.001, Kruskal-Wallis test) (Fig. 3Aa). The fraction of proximal SVs that are tethered and the fraction of proximal SVs that are multiply tethered (three or more tethers) were very significantly decreased in Munc13 DKO synapses (from 0.85 ± 0.04 to 0.57 ± 0.08 and from 0.59 ± 0.06 to 0.13 ± 0.03, respectively, means SEM; *P* < 0.001 in both cases, χ^2^ test) (fig. S5, Ca and Cb). A large fraction of proximal SVs did not harbor any tethers. Accordingly, the distribution of the number of tethers per SV is strongly shifted toward zero to two tethers per SV (fig. S5Ba).

In SNAP25 KO synapses, the mean number of tethers per proximal SV (Fig. 3Aa), the fraction of proximal SVs that were tethered (fig. S5Ca), and the fraction of proximal SVs that exhibited multiple (three or more) tethers (fig. S5Cb) were not altered in comparison to SNAP25 WT synapses (3.2 ± 0.3, 0.79 ± 0.05, and 0.55 ± 0.08, respectively, means ± SEM). However, SNAP25 KO synapses exhibited an increase in the fraction of SVs with only few (one to two) tethers (fig. S5Bb) and a significant decrease in the number of tethers per tethered SV (*P* = 0.015, Kruskal-Wallis test) (fig. S5Cc).

We also confirmed that in WT, the tether length is correlated and the number of tethers inversely correlated with the SV distance to the AZ membrane (both *P* < 0.001, Pearson correlation) (fig. S5A) (*22*, *50*). In addition, we found that in WT, the number of short tethers per SV containing at least one short tether was 1.9 ± 1.1 (means ± SD) and the number of intermediate tethers containing at least one intermediate tethers was 1.8 ± 1.1 (means ± SD) (fig. S5D).

Coapplication of PDBu and the PKC inhibitor Ro31-8220 to plain (untreated) synapses increased the number of tethers per proximal SV, the fraction of tethered proximal SVs, and the fraction of multiply tethered proximal SVs (2.0 ± 0.2, *P* < 0.001, Kruskal-Wallis test; 0.78 ± 0.09, *P* = 0.021, χ^2^ test; and 0.30 ± 0.06, *P* = 0.027, χ^2^ test, respectively; means ± SEM, all in comparison to plain) (Fig. 3Ab and fig. S5, Cd and Ce, respectively). PDBu alone, however, did not significantly increase the number of tethers per proximal SV either in the presence or in the absence of extracellular Ca^2+^ (Fig. 3, Aa and Ab). The mean values of WT and SNAP25 WT synapses (Fig. 3Aa) were similar to each other but different from those of plain synapses (Fig. 3Ab), arguing that this difference is likely a consequence of differing sample preparation and imaging conditions (see Materials and Methods).

In line with our observation that PDBu treatment of synapses lacking Munc13 did not rescue the changes in SV distribution and tether length (Fig. 2, A and C, and fig. S4, D and E), we found that the decrease in the mean number of tethers per proximal SV (Fig. 3Aa), the fraction of tethered SVs, and the fraction of SVs that exhibited multiple tethers seen in absence of Munc13 (fig. S5, Ca and Cb) were not reverted by PDBu.

In contrast, PDBu treatment of synapses lacking SNAP25 very significantly increased the number of tethers per SV (3.7 ± 0.3, *P* = 0.003, Kruskal-Wallis test) (Fig. 3Aa). This increase was mostly caused by an increased fraction of proximal SVs with multiple tethers (0.64 ± 0.07, *P* = 0.003, χ^2^ test) (fig. S5Cb), and it reverted the peak at one to two tethers per SV observed in the distribution of the number of tethers per SV in SNAP25 KO tomograms (fig. S5Bb). This finding is unexpected, given that PDBu treatment of SNAP25 KO synaptosomes did not rescue the shift of SVs to larger distances from the AZ membrane (Fig. 2, Ac and Ad, and fig. S4D) nor the tether length (Fig. 2, Cc and Cd, and fig. S4E).

Together, these results indicate that SNAP25 increases the number of tethers per tethered SV, while Munc13, and, to a lesser extent, PKC inhibition, increases both the fraction of tethered SVs and the number of tethers. The effect of Munc13 had a wider range and was stronger than that of SNAP25. In the absence of SNAP25, PDBu treatment caused an increase in the number of tethers per SV without affecting tether length, just like the coapplication of PDBu and PKC inhibitor Ro31-8220 to WT synapses did. Thus, both conditions decouple the inverse relationship between tether length and the number of tethers per SV that we observed before (*21*). This argues that SNAP25 is not necessary for the PDBu-induced increase in the number of tethers, but it is necessary for the formation of short tethers.

### SNAP25, PDBu, and PKC decrease the proximal SV connectivity

We previously found that tethering alterations observed upon certain genetic manipulations are correlated with modifications of SV connectors (bridges interlinking SVs) (*22*, *50*). Therefore, we investigated whether proximal SV connectors contribute to the SV tethering states defined above.

The connectors were visualized and automatically detected using the same procedure we used for tethers (Fig. 3C). The number of connectors per proximal SV was significantly decreased by PDBu treatment of WT synapses in the presence of extracellular Ca^2+^ and significantly increased in SNAP25 KO samples (from 6.2 ± 0.4 to 2.7 ± 0.4, means ± SEM; *P* < 0.001 in both cases, Kruskal-Wallis test) (Fig. 3Ba). However, neither the removal of Munc13 nor the application of PDBu on Munc13 DKO or SNAP25 KO synapses caused significant changes in the number of connectors per proximal SV (Fig. 3Ba). Furthermore Ro31-8220 + PDBu induced a significant increase in the number of connectors per proximal SV (from 2.0 ± 0.1 to 2.7 ± 0.3, means ± SEM; *P* = 0.034, Kruskal-Wallis test), but PDBu alone did not, both in the absence of extracellular Ca^2+^ (Fig. 3Bb).

We proceeded to determine the locus of the observed SV connectivity changes. We found that the PDBu- and Ro31-8220–induced alterations were highly significant for SVs located at 5 to 10 nm from the AZ membrane (*P* < 0.001 in both cases, Kruskal-Wallis test) (fig. S6, Aa and Ac), whereas the increase in SNAP25 KO synapses affected SVs within 5 to 10 and 10 to 20 nm (*P* = 0.010 and *P* = 0.009, respectively, Kruskal-Wallis test) (fig. S6Ab). Furthermore, despite the lack of a PDBu effect on the number of connectors per SV for all proximal SVs taken together in the absence of extracellular Ca^2+^ (Fig. 3B), there was a significant decrease for SVs within the 10- to 20-nm region upon PDBu and calphostin C + PDBu treatments (*P* = 0.002 and *P* = 0.003, respectively, Kruskal-Wallis test) (fig. S6Ac).

In sum, connector properties followed a pattern distinct from the one observed for tethers. Namely, the activity of the proteins SNAP25 and PKC, as well as treatment by PDBu all decrease the number of connectors between SVs, while the presence or absence of Munc13 has no effect on connectors. All three factors showed a significant connectivity decrease for SVs located 5 to 10 nm to the AZ, indicating that changes in SV connectivity preferentially affect SVs in the intermediate state.

### Munc13 and SNAP25 deletion affects SV size

We found that the radii of proximal SVs were significantly increased in Munc13 DKO and SNAP25 KO synapses (*P* < 0.001 in both cases, *t* test) (fig. S6Ba), in agreement with previous observations in Munc13- and SNAP25-deficient synapses from HPF/FS organotypic slices (*31*). In addition, PDBu decreased the radius of SVs in WT synaptosomes in the presence and absence of extracellular Ca^2+^ and in SNAP25 KO synapses (*P* < 0.001, *P* = 0.003 and *P* = 0.019, respectively, *t* test), but not in Munc13 DKO synapses (fig. S6, Ba and Bc). This phenomenon did not exclusively affect proximal SVs but persisted for all analyzed SVs (within 250 nm to the AZ membrane) (fig. S6Bb).

Therefore, among the conditions tested, smaller SV size correlated with treatments that increase neurotransmitter release and larger SVs with conditions known to disturb neurotransmitter release. The precise mechanisms by which SV size and functional neurotransmitter release properties might be linked are unclear (*31*).

### Structural features of synapses from intact neurons are consistent with synaptosomes

We pursued two strategies to image synapses of dissociated rat hippocampal cultures. First, we searched for synapses in regions distant from cell bodies that were sufficiently thin to allow direct cryo-ET imaging (roughly up to 500 nm). These thin regions contained predominantly nonsynaptic axonal boutons, as observed previously (*42*).

Second, we used both lamella and wedge focused ion beam (FIB) milling geometries at cryo-conditions (cryo-FIB) (fig. S7A) (*51*–*53*). However, these procedures suffer from synapse-specific difficulties such as long culturing times on EM grids, the requirement to optimize culture density to maintain stability of milled regions while insuring proper vitrification, and FIB-induced material redeposition (*54*). All these factors together severely limited the number of synapses that could be located and imaged by cryo-ET, resulting in a total of five tomograms.

We checked that neurons were properly vitrified and had a healthy appearance (fig. S7B and movie S3) and ensured that pre- and postsynaptic membranes formed an extended synaptic cleft of approximately 25 nm in width, as observed before in vitrified synapses (*43*, *55*). This was necessary to properly identify synapses because neurons in culture often have nonsynaptic axonal boutons that contain many SVs and have their plasma membrane closely apposed to a different process at a distance too small for a synaptic cleft (*42*, *56*).

The SV organization, the lack of extended membrane contacts between SVs and the AZ, connectors, and tethers in neuronal cultures were very similar to those observed by cryo-ET in synaptosomes (fig. S7B) (*42*, *43*, *57*, *58*). Quantitative analysis of the overall SV distribution relative to the AZ membrane showed a well-defined peak in the proximal and a minimum in the intermediate zone (fig. S7C). Furthermore, the distributions of proximal SV distances to the AZ membrane and tether lengths were consistent with those of synaptosomes (Fig. 1, C to E).

In addition, we found that the number of short tethers per SV containing at least one short tether was 1.5 ± 0.7 (means ± SD) and the number of intermediate tethers containing at least one intermediate tethers was 2.0 ± 0.9 (means ± SD), similar to the values we obtained for synaptosomes. Therefore, our nanoscale characterization of SV organization and tether length did not show any difference between synapses of intact neurons and synaptosomes.

### Fitting atomic models supports identification of tethers formed by Munc13 and SNARE complex

Biochemical and structural investigations showed that Munc13 and the SNARE complex can bind two lipid membranes simultaneously (*16*, *59*). We therefore examined whether the tethers we detected in our tomograms contain these proteins, by rigid body fitting of the relevant currently available atomic models into the tethers.

**Fig. 4.**
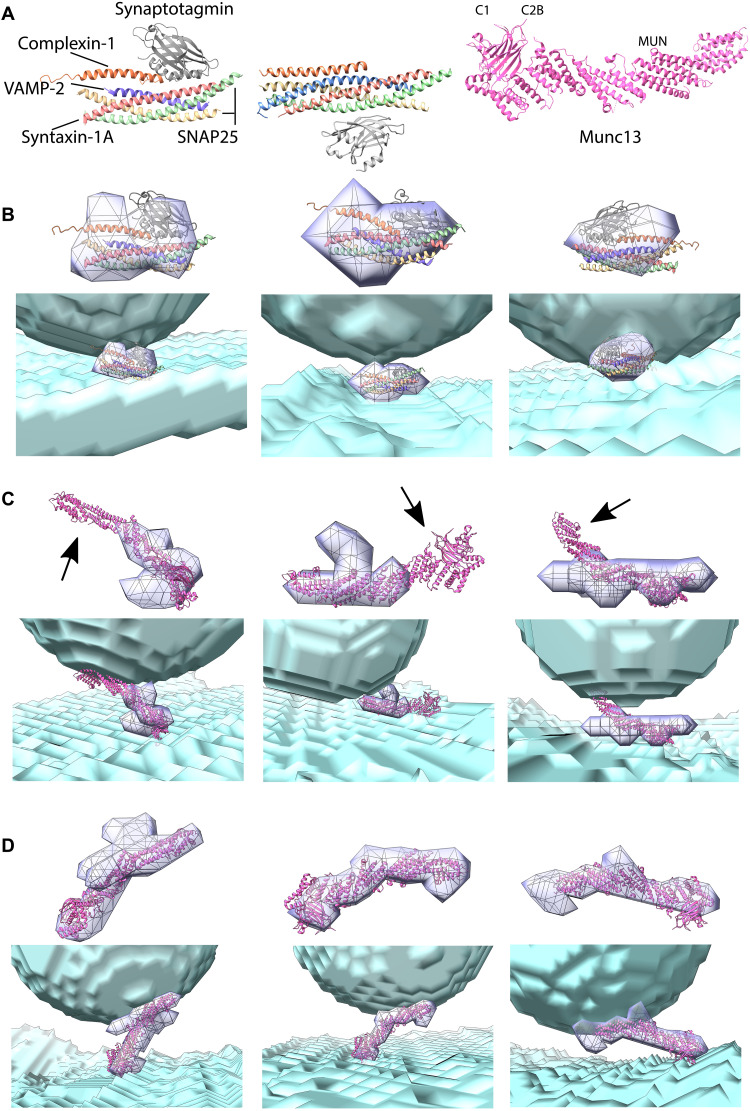
Fitting atomic models supports identification of tethers. (**A**) Atomic models of the SNARE complex (PDB ID: 5w5d; left), (combination of PDB IDs: 1KIL and 5CCH; middle), and Munc13 C1, C_2_B, and MUN domains (PDB ID: 5ue8; right). (**B**) Examples of the primed SNARE complex fitting in short tethers. (**C**) Examples of intermediate tethers where the Munc13 C_1_C_2_BMUN model cannot fit. Arrows point to regions of the model that do not fit into tethers. (**D**) Examples of tethers where the Munc13 C_1_C_2_BMUN model can fit. (B to D) Left: Examples from the WT. Middle: From SNAP25 WT (B) and from SNAP25 KO (C and D). Right: From intact neurons. Gray wire meshes and semitransparent blue-violet volumes show segmented tethers, while the SVs and plasma membrane are shown as semitransparent cyan-blue volumes. In each panel, the same fit is shown without (above) and with the SVs and plasma membrane (below).

First, we used two different atomic models of the SNARE complex, which are thought to link primed vesicles to the AZ membrane. Both models comprise one SNARE motif each of vesticle-associated membrane protein (VAMP2) and Syntaxin 1a and two SNARE motifs of SNAP25, together with a helical fragment of Complexin 1 and the C2B domain of Synaptotagmin 1 [Protein Data Bank (PDB) IDs: 1KIL (*60*), 5CCH (*61*), and 5w5d (*62*)] (Fig. 4A). The two models differ in the position of the C2B domain: In one case, it binds the SNARE complex via the well-established “primary” and, in the other, via the proposed “tripartite” interface (*63*). The four SNARE motifs form a parallel α helix bundle with their C-terminal ends positioned close to their vesicle (VAMP2) and plasma membrane–associated domains (Syntaxin 1a and SNAP25). Both models necessitate a short distance between the SNARE complex–bound SV and the plasma membrane because the C-terminal ends of all four SNARE motifs are very close to their respective membrane anchors.

Considering tethers in both segmented and grayscale density forms, we found that both SNARE complex models fit well into many short tethers in synaptosomes and intact neurons (Fig. 4B). These tethers properly accounted for the distance between the SV and plasma membranes and contained a lateral density that was adequately placed and sufficiently large to account for the SNARE helices. The model orientation was to a large extent constrained by positioning C termini of the model helices close to the tether-lipid contacts and the rest of the model along the tether lateral density. Other short tethers were large enough to accommodate the primed SNARE complex, but the distance between the SVs and plasma membranes was too long, or they contained an extra density that might correspond to additional protein(s).

The second model we used is the largest currently available atomic model of a Munc13 fragment, comprising C_1_, C_2_B, and MUN domains and covering 55% of the entire Munc13 sequence [PDB ID: 5ue8 (*64*)]. Because Munc13 has a rod-shaped MUN domain that is flanked by the lipid-binding C_2_B and C_2_C domains (Fig. 4A) and the MUN domain is homologous to tethering factors in other systems, Munc13 was hypothesized to form tethers (*4*, *65*, *66*).

A fit was deemed acceptable if the region from the C_2_B domain to the C-terminal end of MUN domain could be placed within the segmented tether. We found that this Munc13 model could fit into many intermediate and long tethers. This might be unexpected, considering that the distance between the vesicle and the plasma membrane binding regions of Munc13 is at least 13.0 nm, as determined by measuring the shortest distance between the C_2_B domain and the C-terminal end of the MUN domain of the Munc13 atomic model. However, these tethers contained extended density along the plasma and/or vesicle membranes that made fitting the Munc13 C_1_C_2_BMUN model possible (Fig. 4D). Nevertheless, we found that 19 of 49 intermediate tethers in SNAP25 KO synapses were too small to accommodate the Munc13 C_1_C_2_BMUN model (Fig. 4C). Furthermore, the model could not fit 18 of 61 tethers in WT and Munc13 DHet synapses and 16 of 31 tethers in intact neurons. The fact that the Munc13 C_1_C_2_BMUN model only covers 55% of the Munc13 sequence and does not include binding partners increases the significance of this finding.

In sum, short tethers were sufficiently large to accommodate the primed SNARE complex, and the Munc13 C_1_C_2_BMUN domain fit into many intermediate tethers. However, a significant number of intermediate tethers could not accommodate any of these models. Because these tethers were observed in WT, Munc13 DHet, SNAP25 KO, and intact neurons, they are most likely stable and of physiological relevance, and at least some of them do not contain SNAP25.

## DISCUSSION

### Methodological considerations

Over the past three decades, we have achieved a very detailed understanding of the molecular, morphological, and functional aspects of the neurotransmitter release process and the underlying SV cycle. This progress is mainly owed to genetically modified model organisms lacking defined protein components of the SV cycle. However, gaining detailed insight into the complex molecular machinery that operate the SV trafficking steps preceding fusion, characterizing their precise composition, localization, and interrelation in synapses, and pinpointing the precise roles of the key molecular players that conduct separate SV trafficking tasks have remained exceedingly difficult, which led to many controversies in the field. Arguably, the biggest methodological problem in this context has been that only EM approaches provide the necessary resolution to visualize the distribution of SVs at individual AZs with nanometer precision. Most such approaches require postfixation dehydration of samples, heavy-metal staining of membrane and cytomatrix components, and embedding in plastic resin for ultramicrotomy. This allows the in-depth characterization of the SV organization relative to the AZ under different experimental conditions and the indirect immunodetection of proteins, but artifacts introduced by the individual processing steps cannot be avoided, so that proteins cannot be seen directly with appropriate stringency.

Cryo-ET provides the most promising way forward in this regard. It permits the visualization of synaptic protein complexes in the vitrified frozen hydrated state, without the use of heavy metals to enhance membrane contrast. It enables the visualization of SVs and the AZ plasma membrane–bound complexes, thus allowing insights into the native molecular organization of synaptic proteins in minimally perturbed synapses at a single-nanometer scale.

Our observation that Munc13 and SNAP25 are needed for SV localization at <10 nm and <5 nm to the AZ membrane, respectively, agrees qualitatively with the SV distribution seen in the same mutants obtained by ET of HPF/FS samples (*30*, *31*). However, the corresponding distances are shorter with HPF/FS. Thus, SVs in apparent membrane contact with the AZ membrane in HPF/FS, considered “docked” in a SNARE-dependent and molecularly primed state, correspond to SVs located <5 nm but not touching the AZ membrane in cryo-ET. SVs upon Munc13 DKO accumulate at 8 to 10 nm from the AZ membrane in HPF/FS-ET and at >10 nm in cryo-ET. This discrepancy is likely due to single-nanometer-scale rearrangements caused by dehydration and heavy metal staining of HPF/FS (*67*, *68*). In HPF/FS, membrane-proximal vesicles that accumulate at stereotypic distances upon deletion of SNAREs or Munc13s are inferred as being “tethered” (*31*, *69*, *70*). Despite this methodology-based discrepancy, the relative increases in the distance separating SVs from the AZ membrane upon deletion of SNAP-25 or Munc13s are notably comparable between HPF/FS and frozen-hydrated preparations. Further, the increase in SV size upon Munc13 and SNAP25 deletion as observed in organotypic slices prepared by HPF/FS (*31*) agrees with our present cryo-ET results from synaptosomes. This supports our earlier conclusion that despite differences, it is possible to establish a correspondence between the data from vitrified and HPF/FS samples and that the latter provides a better preservation than chemical fixation-based preparations (*18*).

Regions of vitrified dissociated neuronal cultures where most synapses are located are mostly too thick for cryo-ET imaging (*42*), thus necessitating sample thinning by cryo-FIB milling. We observed that the structural features in synapses of intact neurons were consistent with results from synaptosomes and that membrane contacts between SVs and the AZ were extremely rare in synaptosomes and synapses from intact neurons, in agreement with previous cryo-ET assessments (*21*, *42*, *43*, *57*, *58*). Together, the correspondence between our cryo-ET results from synaptosomes with the HPF/FS results from organotypic slices and the agreement between cryo-ET date from synaptosomes and intact synapses provide orthogonal validations of our synaptosome model to study the molecular architecture of synapses.

Here, we combined genetic approaches [knockout mice for key regulators of AZ organization (RIMs), SV priming (Munc13s), and fusion (SNAP25)] with cryo-ET of synaptosomes. While RIM cDKO abolishes expression of all relevant RIMs (*46*) and Munc13 DKO abrogates both evoked and spontaneous release (*6*, *7*), compensatory effects cannot be excluded in SNAP25 KO synapses because SNAP23 supports asynchronous release and a low level of spontaneous release remains despite severe release impairment (*37*, *71*).

### Potential molecular identity and function of SV tethers

We visualized complexes involved in SV priming and neurotransmitter release at a single-nanometer resolution in their native composition, conformation, and environment, within entire presynaptic terminals. Precise localization, analysis of structural alterations caused by genetic and pharmacological perturbations, and molecular fitting allowed us to identify some of the SV-interacting presynaptic complexes and led us to propose an SV tethering and priming model that combines structural and molecular information.

In line with our previous work (*21*, *22*), we found that tethers, defined structurally as all directly observed bridges linking SVs with the AZ membrane, exhibited a wide, molecular condition–dependent range of lengths, arguing that tethers of different molecular compositions coexist at the synapse. Furthermore, changes in tether length induced by genetic and pharmacological manipulations paralleled those of SV distance to the AZ membrane. Because, under different conditions, SVs assumed markedly different, nonuniform spatial distribution, despite their thermally induced diffusion that renders the distribution more uniform, and because we did not observe other structures that could influence this distribution, our data strongly argue that tethers control the SV distance to the AZ.

We show that RIM isoforms other than RIM1α also contribute to localization of SVs in the proximal zone. This indicates that the partial compensation observed earlier in a subset of synapses lacking RIM1α was due to 1β, 2α, and/or 2β isoforms (*22*). Synapses lacking RIMs showed an absence of structures that could constrain SV distance to the AZ membrane. This is expected because the absence of RIMs reduces Munc13-1 expression levels and RIMs activate Munc13-1 (*8*, *9*).

**Fig. 5.**
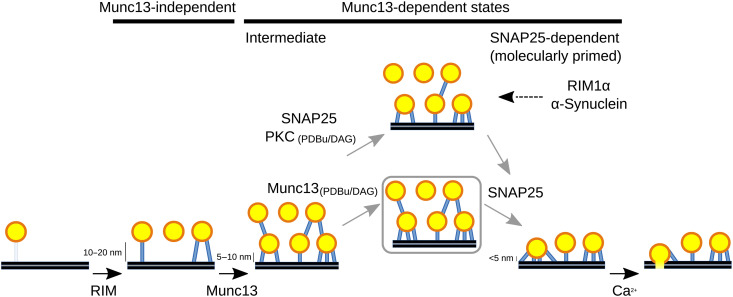
Structural model shows roles of Munc13 and SNAP25 in SV tethering and priming. All SV distances to the AZ membrane and the molecular assignments result from this study, except the influence of RIM1α and α-synuclein on SV connectors, which was reported earlier (*22*, *50*) and is shown here to provide a complete model. The gray box and gray arrows indicate possible separate contributions of PDBu and SNAP25 to the transition from the intermediate to the SNAP25-dependent state. The connector-mediated process is shown above. The model is not drawn to scale to emphasize that SVs are located at different distances to the AZ membrane.

Our data strongly argue that Munc13s (Munc13-1 and Munc13-2) and SNAP25 act to reduce the proximal SV distance to the AZ membrane and tether length with a precision of a few nanometers, allowing us to distinguish a Munc13-independent SV tethering state and two Munc13-dependent states, namely, the SNAP25-dependent and the intermediate state (Fig. 5). The alterations of the SV distribution, tether length, and the number of tethers per SV caused by the deletion of Munc13 were more pronounced than those of SNAP25, arguing that the tethering function of Munc13 is upstream from SNAP25. Therefore, we conclude that regarding SV progression toward fusion, the Munc13-independent state is the most upstream, followed by the intermediate and the SNAP25-dependent states.

These conclusions agree with the known function of Munc13 in facilitating the SNARE complex assembly and with the currently accepted intuitive notion that SV progression toward fusion proceeds by reducing the SV distance to the AZ membrane (*1*, *4*), showing the predictive power of our approach.

Fitting atomic models of known molecular candidates for SV tethers, constrained by the known orientation and location of interacting partners, allowed us to identify tethers that could accommodate one of the models, with or without additional proteins. Given the large morphological difference between the available models, this permitted detection of tethers that could very likely accommodate either the SNARE complex or Munc13.

The shape and orientation of the majority of intermediate tethers agreed with the atomic model of the Munc13 C_1_C_2_BMUN fragment, previously proposed to bridge SV and plasma membranes (*64*, *66*). Given that it was earlier argued that neuronal SNAP25 paralogs are unlikely to significantly compensate for a loss of SNAP25 (*31*, *71*), most of the intermediate state SVs are likely SNAP25 independent. Considering that the intermediate tethers required Munc13, our data suggest that most intermediate tethers contain Munc13.

The SNAP25-dependent state likely corresponds to the molecularly primed state because it requires Munc13 and SNAP25, proteins necessary for functional priming (*6*, *7*, *37*). We showed that short tethers strongly depend on the presence of SNAP25, an obligatory component of the SNARE complex, and that their shape and orientation are consistent with the atomic model of the primed SNARE complex. This argues that short tethers likely comprise the SNARE complex, in agreement with the fundamental role the SNARE complex plays in membrane fusion (*3*–*5*). This finding does not preclude the presence of partially zippered SNARE complexes because these are likely morphologically similar to the primed SNARE complex and thus hardly distinguishable in our tomograms.

Furthermore, our data showed that in the presence of Munc13 and SNAP25, DAG/PDBu promotes the transition to the SNAP25-dependent state. In the absence of Munc13, PDBu did not alter the SV localization or tethering, arguing that PDBu directly affects Munc13, as shown before (*12*). In synapses lacking SNAP25, PDBu increased the number of tethers without promoting the formation of short tethers and the transition to the SNAP25-dependent state. This may indicate that SV priming proceeds by a DAG/PDBu-Munc13–dependent formation of intermediate tethers, followed by a constitutive formation of short tethers that requires SNAP25 and possibly other SNARE proteins and Munc18.

On the basis of our structural data, we propose a sequential SV tethering and priming model comprising the following steps (Fig. 5). (i) RIM family is responsible for the accumulation of SVs in the proximal zone (within 45 nm to the AZ membrane) in a Munc13-independent manner. (ii) Munc13-dependent intermediate tethers are necessary to localize SVs <10 nm to the AZ membrane to the intermediate SV tethering state. (iii) SNAP25-dependent short tethers are needed to localize SVs <5 nm to the AZ membrane into the SNAP25-dependent (molecularly primed) state. The transition to this state is facilitated by DAG/PDBu and requires both the upstream function of Munc13 and SNAP25. (iv) Ca^2+^ influx may lead to vesicle fusion.

### Possible implications for current models of presynaptic function

While we could clearly distinguish three SV tethering states, the complete picture might be more complex. We detected intermediate tethers that could not accommodate any of the previously proposed atomic models that may bridge SV and plasma membranes (Munc13 and SNARE complexes). They were too small to be reconciled with the Munc13 C_1_C_2_BMUN model, and the distance between their SV and plasma membrane lipid binding regions was too large for the SNARE complex models. Because at least some of these tethers did not contain SNAP25, we speculate that full-length native Munc13 can adopt a more compact structure than the Munc13 C_1_C_2_BMUN model. The flexibility of Munc13 observed in purified and reconstituted systems (*64*, *66*, *72*, *73*) is not sufficient to explain the Munc13-incompatible intermediate tethers. Alternatively, these tethers could be formed by a membrane-bridging complex of a size between that of SNARE complex and Munc13 that does not contain SNAP25 or Munc13 but may contain other proteins, such as Munc18-1 as proposed earlier (*1*, *74*), or a larger SNAP25 paralogue. Although these alternative molecular compositions cannot be verified in our study, our data argues that tethers of different composition or conformation are indeed present at the synapse. The observed broad shape of tether length distribution peaks (fig. S1, Dc and Ec) was likely caused by the combination of tether variability and a possibility that different lipid binding modes of the same tether type yield different lengths.

Recent studies reported a trimeric or hexameric organization of Munc13 in 2D crystals of a large portion of Munc13-1 (*73*) and a hexameric organization of SNAP25-dependent tethers in dissociated neuronal cultures (*75*). We observed such organization neither in neuronal cultures nor in synaptosomes but found that the mean number of SNAP25-dependent (short) and Munc13-dependent (intermediate) tethers was between 1.5 and 2.0 in both preparations. We observed individual tethers directly, without averaging, and considered an SV-rich bouton a presynaptic terminal only if it was apposed to a clearly identified postsynaptic terminal over a synaptic cleft of ∼25 nm in width, as described before (*42*, *43*).

It was previously proposed that distinct subpools of functionally primed SVs exist (*76*, *77*) that exhibit differences in release probability. In this context, DAG/PDBu increases the fraction of high-release probability (“superprimed”) SVs relative to the low-release probability primed SVs at the calyx of Held synapses (*78*). On the basis of our data, we hypothesize that at least some SVs in the SNAP25-dependent SV tethering state correspond to the superprimed functional state. Among the molecularly primed SVs, a transition from low- to high-release probability state may be achieved by the addition of new SNARE complex–dependent short tethers that may help to pull SVs and AZ membranes together. This is consistent with our earlier hypothesis that a higher number of tethers facilitates SV progression toward fusion (*18*, *21*) and the proposal that small changes in the number of SNARE complexes can regulate priming and release (*79*). While the intermediate-state SVs are not molecularly equipped to fuse in response to a single action potential, they may rapidly transition into the fusion-competent SNAP25-dependent (molecularly primed) state during synaptic activity (*69*, *77*, *80*–*83*).

We observed that PDBu, SNAP25, and PKC, but not Munc13, decrease the connectivity between proximal SVs, most prominently for SVs located 5 to 10 nm to the AZ membrane. The effects of PDBu were reported to be mediated by Munc13- and PKC-dependent pathways (*12*, *48*, *64*, *84*–*88*). This argues that connectors are relevant for the transition from the intermediate to the SNAP25-dependent state. Further support for this statement is provided by our observations that PKC might affect SV localization by a tether-independent mechanism and that inhibiting PKC perturbs the SV transition to priming. Therefore, our data suggests that two SNAP25-dependent molecular processes are involved in this transition, while the PDBu-Munc13 process is mediated by tethers, and the PDBu-PKC process is mediated by connectors (Fig. 5).

Rab3, an SV-associated protein, RIMs, and Munc13 were early on implicated in functionally superpriming SVs. We previously detected an increased connectivity in synapses lacking RIM1α and in synapses that overexpressed human α-synuclein on a synuclein null background (*22*, *50*, *89*). This suggests that RIMs, Rab3, and possibly synucleins may be involved in the connector-mediated process. Furthermore, RIMs were shown to be important in presynaptic plasticity, while PDBu-induced and other forms of plasticity were associated with Rab3 superpriming (*78*, *90*). Therefore, we propose that the tether-mediated priming process (Munc13- and SNAP25-dependent) is modulated by the connector-mediated process (SNAP25-dependent and Munc13-independent) and that the latter is relevant for presynaptic plasticity, thus extending our earlier hypothesis (*18*).

The relation between SNAP25 and PKC in the connector-mediated process is unclear. The possibility that PDBu-induced PKC phosphorylation of SNAP25 potentiates synaptic release was recently contested (*91*–*93*). Alternatively, PKC and SNAP25 could act independently from each other to decrease connectivity, with SNAP25 being associated with SVs in a way that blocks or competes with connector formation. Considering that SNAP25 is also needed to form SNARE complexes, this would require a high concentration of SNAP25 molecules in the proximal zone, which is consistent with the previously reported very high copy number of SNAP25 within the presynapse (*94*). Another possibility is that PKC phosphorylates Munc18-1, a protein known to bind directly to the SNARE complex (*95*, *96*).

The opposite roles of SNAP25, increasing the number of tethers and decreasing the number of connectors, present an inverted picture in comparison to synucleins, previously shown to negatively affect tethers and support connectors (*50*). This suggests that a concerted, differential regulation of tethers and connectors represents a specific pattern that influences the SV dynamics in the proximal zone.

Last, we previously described trans-synaptic subcolumns, tripartite protein assemblies that contain tethers and provide a structural link between SVs and postsynaptic receptors, and hypothesized that they ensure efficient synaptic transmission by colocalizing SVs and postsynaptic receptors across the synaptic cleft (*23*). We showed that subcolumns cluster to form large, nonuniform molecular assemblies, which may be closely related to synaptic nanocolumns that were previously described by super-resolution fluorescence imaging (*23*, *97*, *98*). Our current findings raise the possibility that connectors interlink subcolumns to form structuraly defined synaptic nanocolumns, which organize synaptic transmission. Because these assemblies are intermixed with other complexes, they cannot be isolated and have to be investigated at molecular resolution in situ, thus making our cryo-ET–based approach uniquely suited to achieve this objective. Therefore, our results extend the notion of molecular complexes serving as basic functional modules (*99*) and present an example of a cellular process carried out by a concerted action of multiple spatially separated and molecularly diverse complexes comprising a large, nonperiodic, protein assembly.

Together, our data suggest that structurally defined SV tethers comprising Munc13 and the SNARE complex differentially confine SVs with a single-nanometer precision. These results define sequential SV tethering states that precede neurotransmitter release and provide molecular mechanisms that precisely localize SVs and explain their observed nonuniform spatial distributions at presynaptic release sites.

## MATERIALS AND METHODS

### Munc13- and SNAP25-deficient mouse breeding

Munc13-deficient mice were bred with permission of the Niedersächsisches Landesamt für Verbraucherschutz und Lebensmittelsicherheit (LAVES; 33.19.42502-04-15/1817). All mice were kept according to the European Union Directive 63/2010/EU and ETS 123. They were housed in individually ventilated cages (type II superlong, 435-cm^2^ floor area; TECHNIPLAST) under a 12-hour light/dark cycle at 21° ± 1°C with food and water ad libitum, and the health status of the animals was checked regularly by animal care technicians and a veterinarian. Hippocampal organotypic slice cultures were prepared from C57 B6/N WT animals on P0 and from Munc13- and SNAP25-deficient and littermate control animals at E18 due to perinatally lethal phenotypes (*7*, *37*). E18 mice lacking Munc13-1 and Munc13-2 (Munc13^−/− −/−^, denoted Munc13 DKO) (*6*, *7*) and control littermates were generated by crossing Munc13-1^+/−^;Munc13-2^+/−^ with Munc13-1^+/−^;Munc13-2^−/−^ or Munc13-1^+/−^;Munc13-2^+/−^ mice. Cultures from control littermates with the genotypes Munc13-1^+/−^ Munc13-2^+/−^ (Munc13 DHet) and from P0 control C57 B6N WT animals (M13 WT) were prepared on the same day. E18 mice lacking SNAP25 (SNAP25^−/−^, denoted SNAP25 KO) were generated by cross-breeding SNAP25^+/−^ mice. Control littermates were WT for the SNAP25 allele (SNAP25 WT). We used and pooled data generated from female and male mice since loss of Munc13 or SNAP25 affects synaptic transmission equally in neurons from both genders. That said, we cannot exclude the possibility that subtle changes in the organization of vesicle pools may exist between male and female neurons in WT cultures. Analyses were performed 3 to 5 weeks after culturing. On the day of experiment, slices from animals of the same genotype from cultures prepared on the same day were pooled to get sufficient material for synaptosome preparation.

### Organotypic slice culture preparation from Munc13- and SNAP25-deficient mice

Hippocampal organotypic slice cultures were prepared from Munc13- and SNAP25-deficient and the corresponding controls mice using the interface method (*100*) as described previously (*31*, *69*). Pregnant females at E18 were anesthetized and decapitated, and embryos were removed by hysterectomy. Pups were decapitated, and hippocampi were dissected in dissection medium [97 ml of Hank’s balanced salt solution (HBSS), 2.5 ml of 20% glucose, and 1 ml of 100 mM kynurenic acid (pH 7.4)]. Three hundred–micrometer–thick hippocampal slices with the entorhinal cortex attached were prepared using a McIlwain tissue chopper. Slices were then transferred onto sterile Millipore membrane confetti pieces on top of six-well membrane inserts in 1.2 ml of culture medium (22.44 ml of double-distilled H_2_O, 25 ml of 2× minimum essential medium, 25 ml of Basal Medium Eagle (BME), 1 ml of GlutaMAX, 1.56 ml of 40% glucose, and 25 ml of horse serum). Residual dissection medium was removed using a P200 pipette. A maximum of four hippocampal slices were cultured per membrane insert at 37°C and 5% CO_2_. Slice culture medium was changed 24 hours after preparation and then two to three times per week. Slices were cultured for 3 to 5 weeks, allowing them to recover from the dissection trauma and develop mature synapses (*31*, *101*).

### Synaptosomal preparation from Munc13- and SNAP25-deficient slice cultures

Munc13- and SNAP25-deficient and the corresponding control synaptosomes were prepared from day in vitro (DIV) 28 to 30 hippocampal organotypic slices. The incubation buffer was removed, and the slices were briefly rinsed with Tyrode’s buffer [120 mM NaCl, 3 mM KCl, 1.25 mM MgCl_2_, 1.25 mM CaCl_2_, 0.5 mM NaH_2_PO_4_, 25 mM Hepes, and 30 mM d-glucose (pH 7.4)] at room temperature. They were scooped with a brush and dropped in a glass tube containing the homogenization buffer [HB; 0.32 M sucrose and one tablet of cOmplete mini EDTA-free protease inhibitor cocktail (Roche; 10 ml; pH 7.4)] at 4°C (50 μl of buffer for each slice). Slices were homogenized in a teflon glass homogenizer applying one stroke at 100 rpm and seven strokes at 700 rpm. The homogenate was centrifuged at 2000*g* for 2 min (twice), the pooled supernatants were centrifuged for 12 min at 9500*g*, and the pellet (P2) was resuspended in Ca^2+^-free Hepes-buffered medium [HBM; 140 mM NaCl, 5 mM KCl, 5 mM NaHCO_3_, 1.2 mM NaH_2_PO_4_-H_2_O, 1 mM MgCl_2_-6H_2_O, 10 mM glucose, and 10 mM Hepes (pH 7.4)], yielding crude synaptosomal fraction (all at 4°C). The protein concentration was measured using the Bradford Assay (Bio-Rad) on an Amersham Biosciences Ultrospec 3100 Pro Spectrophotometer (GE Healthcare) and diluted to 0.3 to 0.5 mg/ml. The fraction was centrifuged at 10,000*g* for 10 min, and the pellet was stored on ice.

One hour before vitrification, the pellet containing the synaptosomes was thoroughly and carefully resuspended with warm HBM + 1.2 mM CaCl_2_ and was incubated at 37°C. Some synaptosomes (as indicated in Results) were treated with 1 μM PDBu in dimethyl sulfoxide (final concentration) for 15 min.

### Neocortical synaptosomal preparation

Cerebrocortical synaptosomes were extracted from 6- to 8-week-old male Wistar rats as described previously (*22*, *40*, *102*) in accordance with the procedures accepted by the Max Planck Institute for Biochemistry. Briefly, animals anesthetized with chloroform were decapitated, and the cortex was extracted and homogenized in HB [0.32 M sucrose, 20 mM dithiothreitol, and one tablet of cOmplete mini EDTA-free protease inhibitor cocktail (Roche; 10 ml; pH 7.4)] with up to seven strokes in a Teflon glass motor driven homogenizer at 700 rpm. The homogenate was centrifuged at 2000*g* for 2 min (twice), the pooled supernatants were centrifuged for 12 min at 9500*g*, and the pellet (P2) was resuspended in Ca^2+^-free HBM (all at 4°C), yielding crude synaptosomal fraction. The suspension was loaded onto Percoll (GE Healthcare) gradient (3, 10, and 23%, all in 0.32 M sucrose solution), and the maximum suspension volume was 1 ml per gradient. The gradients were centrifuged at 25,000*g* at 4°C for 6 min, and the 10/23% interface was retrieved with a Pasteur pipette and diluted in 50 ml of HBM. Percoll was removed by an additional washing step with HBM by centrifugation for 10 min at 22,000*g*. As we could detect and image healthy synaptosomes from the crude synaptosomal fraction similar to the ones prepared by Percoll gradient centrifugation, we decided to omit Percoll gradient for synaptosomes intended for cryo-ET. Percoll gradient purified synaptosomes were used for all glutamate release assays. In all cases, samples were diluted in HBM to a final concentration of 0.7 mg/ml, as determined by Protein-UV (Implen NanoPhotometer). Suspension was spun down at 10,000*g* for 10 min (in tubes containing 1 ml), and pellets were kept on ice for a maximum of 4 hours. All steps were performed at 4°C. At no point during this procedure were synaptosomes subjected to hypertonic conditions.

The pellet was thoroughly and carefully resuspended in warm HBM and incubated at 37°C for 1 hour before vitrification. During that time, synaptosomes were treated with 0.1 μM calphostin C for 30 min, 0.1 μM Ro31-8220 for 30 min, and 1 μM PDBu for 5 min, as indicated in fig. S3. The lack of extracellular Ca^2+^ in this preparation is in contrast to the resuspensions in HBM + 1.2 mM CaCl_2_ that were used for synaptosomes from Munc13- and SNAP25-deficient slice cultures.

### Glutamate release assay

The glutamate release assay was performed according to (*103*) with modifications. Briefly, the synaptosomal pellet obtained using the Percoll gradient procedure was resuspended in Ca^2+^-free HBM + bovine serum albumin (BSA) (1 mg/ml) to a protein concentration of 0.7 mg/ml and incubated at 37°C for 60 min.

Nicotinamide adenine dinucleotide phosphate (NADP^+^; 1 mM) and glutamate dehydrogenase (GDH; 50 U/ml) were added 120 s before the measurements started. The fluorescence, resulting from glutamate oxidation by GDH-induced reduction of NADP^+^ to reduced form of NADP^+^, was measured in a spectrometer (Luminescence Spectrometer LS 50 B, PerkinElmer) for 600 or 700 s. Excitation and emission wavelengths were 340 and 460 nm, respectively, and the sample was stirred in glass cuvettes. For calibration, 200 s before termination of the measurement, 2 μM glutamate was added as a reference. The calibration was done for each trace individually.

For measurements shown in fig. S3A, synaptosomes were treated with 0.1 μM calphostin C, 0.1 μM Ro31-8220, and 1 μM bisindolylmaleimide 1 for 30 min and 1 μM PDBu for 5 min. After incubation, synaptosomes were washed by 30-s centrifugation at 13,000 rpm and resuspended in Ca^2+^-free HBM + BSA (1 mg/ml) to a final protein concentration of 0.68 mg/ml. We added 1.33 mM CaCl_2_ at the beginning of the fluorescence measurements. Number of measurements for each condition was 5 to 7.

For measurements shown on fig. S3B, there were no pharmacological treatments performed during the incubation period. We added 0.7 mM CaCl_2_ 100 s after the beginning of the fluorescence measurement. In addition, 1 μM PDBu was added at the beginning (in some cases, PDBu) and was not added in other (control). In both cases, at 40 s after the onset of measurements, synaptosomes were treated with 5 mM KCl and 2.5 to 3 μM ionomycin, or they were not stimulated. Five measurements were done for each condition.

### RIM conditional knockout mice

Conditional RIM1/2^flox/flox^ mice (RIM1^flox^:RIM2^flox^: RIMS1^tm3Sud^/J, RIMS2^tm1.1Sud^/J, The Jackson Laboratory) (*46*) were used for all analyses. Mice were housed under a 12-hour light/dark cycle (light cycle, 7 a.m. to 7 p.m.), in a temperature (22° ± 2°C)– and humidity (55 ± 10%)–controlled environment with food/water ad libitum. All procedures were planned and performed in accordance with the guidelines of the University of Bonn Medical Centre Animal Care Committee and the guidelines approved by the European Directive (2010/63/EU) on the protection of animals used for experimental purposes.

### Dissociated neuronal culture preparation from RIM conditional knockout mice

Mouse cortical neurons were prepared from RIM double floxed embryonic mice (E16 to E18) as previously described (*104*). Briefly, after dissection of cortices from embryonic mice and several rounds of washing with HBSS (Life Technologies), cells were digested with trypsin (0.025 g/ml; Life Technologies) for 20 min at 37°C. After several washing steps with HBSS, the remaining DNA was digested with deoxyribonuclease I (DNase I; 0.001 g/ml; Roche). Cannulas were used to dissociate the tissue, and the suspension was passed through a Nylon cell strainer (100 μm; BD Biosciences). Cells were seeded in a 10-cm dish coated with poly-d-lysine at a density of 1.2 million cells per dish or in a six-well plate coated with poly-d-lysine at a density of 300,000 cells per well. Neurons were cultured in BME (Life Technologies) supplemented with 0.5% glucose (Sigma-Aldrich), 10% fetal bovine serum (Life Technologies), 2% B-27, and 0.5 mM l-glutamine (Life Technologies) or Neurobasal medium (Thermo Fisher Scientific). Cells were maintained at 37°C in 5% CO_2_ until use. All embryos of a single mother were combined to maximize the number of neurons.

### Lentivirus production and infection of RIM double floxed neuronal cultures

Lentiviruses were produced using a second-generation packaging system, as previously described (*105*). Briefly, 3 × 10^6^ human embryonic kidney 293T cells (Clontech) were seeded on a 10-cm cell culture dish and transfected after 24 hours with GenJet transfection reagent (SignaGen). Per dish, 7.5 μg of packaging plasmid (psPax2, Addgene), 5 μg of vesicular stomatitis virus glycoprotein–expressing envelope plasmid (pMD2.G, Addgene), and 4 μg of plasmid of interest [e.g., Cre–green fluorescent protein (GFP)] were used. After 12 hours, transfection medium was replaced with Dulbecco’s modified Eagle’s medium containing GlutaMAX (Invitrogen) supplemented with 10% fetal bovine serum. Transfected cells were incubated for 72 hours to allow the formation of viral vectors. Thereafter, the supernatant was filtered through 0.45-μm polyvinylidene difluoride membrane filters (GE Healthcare) to remove cell debris and other aggregates. To purify the virus, the filtered supernatant was layered on top of OptiPrep density gradient medium (Sigma-Aldrich) and centrifuged at 24,000 rpm for 2 hours at 4°C using a SW-Ti32 swinging bucket (Beckman Coulter). The upper layer was discarded. The OptiPrep Layer, with the viral particles at its upper boundary, was mixed with TBS-5 buffer (containing in 50 mM tris-HCl, 130 mM NaCl, 10 mM KCl, and 5 mM MgCl_2_). Viral particles were pelleted by centrifugation (24,000 rpm for 2 hours at 4°C) and resuspended in TBS-5 buffer. Lentiviruses were stored at −80°C until use. Primary neurons cultured in 10-cm cell culture dishes were transduced with 7 μl of lentiviral suspension per dish at DIV 2 to 6. For the transduction of primary neurons cultured in six-well plates, 3 μl of lentiviral suspension was used per well. The primary cultured neurons were transduced with lentiviruses expressing active or inactive mutant-Cre recombinase to yield RIM double knockout and RIM WT cells. Lentiviral vectors encoding nuclear localization signal (NLS)–GFP–Cre and NLS-GFP-deltaCre were provided by T. Südhof (Stanford University, Stanford, CA).

### Synaptosomal preparation from RIM conditional knockout cultures

RIM double floxed dissociated neuronal cultures (DIV 13 to 16), either treated with (GFP)-Cre or NLS-GFP-deltaCre expressing lentiviral particles at DIV 2 to 6, were briefly rinsed with 2 ml of Tyrode’s buffer at room temperature; the buffer was removed; the dishes were placed on ice and 500 μl of HB [0.32 M sucrose and one tablet of cOmplete mini EDTA-free protease inhibitor cocktail (Roche; 10 ml; pH 7.4)] (at 4°C) was added. The neurons were scraped off and placed in a glass homogenization tube (all at 4°C). Neurons were homogenized by one stroke at low speed (100 rpm) and seven strokes at 700 rpm. The homogenate was centrifuged at 2000*g* for 2 min (twice), the pooled supernatants were centrifuged for 12 min at 9500*g*, and the pellet (P2) was resuspended in Ca^2+^-free HBM (all at 4°C), yielding the crude synaptosomal fraction. The protein concentration was measured using a NanoDrop spectrophotometer (Thermo Fisher Scientific) and diluted with Ca^2+^-free HBM to little below 1 mg/ml. The sample was centrifuged at 10,000*g* for 10 min, and the pellet was kept on ice until vitrification.

The above protocol was used for the data presented here. Despite several attempts to optimize the preparation, it has been extremely challenging to find synaptosomes that could be imaged by cryo-ET, for both RIM cDKO and RIM Ctrl. Less successful variations of this protocol that we explored involved using a filter (5-μm pore size) to remove large chunks (*106*) instead of homogenization and low speed centrifugation, purifying the crude synaptosomal fraction on a 3/10/23% discontinuous Percoll gradient and varying the amount and the concentration of synaptosomes.

### WT dissociated neuronal culture

Primary neuronal culture was prepared according to the Banker method (*107*) modified to grow cells on EM grids (*57*). Gold Quantifoil grids (R1/4, Au 200 mesh, Quantifoil Micro Tools) were additionally coated with a 20- to 25-nm carbon layer (Med 020, Bal-Tec). For sterilization, grids were transferred to four-well dishes (Falcon) and ultraviolet-irradiated for 30 min on a sterile bench. EM grids were coated with poly-l-lysine in a solution (1 mg/ml) in 0.1 M borate buffer (pH 8.5) overnight in the dark and glass bottom dishes for light microscopy. Unbound poly-l-lysine was washed multiple times with autoclaved Milli-Q water, and the grids were soaked in neuronal plating medium until cell seeding.

To make an astroglial cell feeder culture, P1 pups of Sprague-Dawley rats were euthanized according to the guidelines of the Max Planck Institute of Biochemistry. After removing meninges, hippocampi and cortices were dissected and transferred to ice-cold CMF-HBSS-Hepes (calcium- and magnesium-free HBSS with 5% Hepes; Invitrogen). Tissue was minced and digested in 0.25% Trypsin and 0.1% (w/v) DNase I in a 37°C water bath for 15 min and triturated with a 10-ml automated pipette. Cells were passed through a 70-μm cell strainer (Becton Dickinson and Company), centrifuged at 120*g* for 10 min to remove enzymes and lysed cells, and resuspended in glial medium [minimal essential medium with Earl’s salt and l-glutamine, 0.6% d-glucose, penicillin-streptomycin (100 U/ml), and 10% fetal bovine serum; Invitrogen). Glial cells were plated in 75-cm^2^ flasks (Falcon) at a density of 7.5 × 10^6^ cells per flask and incubated in a CO_2_ incubator at 37°C. Medium was exchanged every third day, and flasks were swirled harshly to remove loosely attached cells from the flask surface such as microglia or oligodendrocyte progenitor cells (O2A). Nearly confluent astroglia were harvested by trypsination, centrifuged at 120*g* for 7 min, and resuspended in glial medium for seeding in 6-mm dishes at a concentration of 1.0 × 10^5^ cells per dish. Glial medium was exchanged every 3 to 4 days and replaced by Neurobasal/B27 medium for preconditioning. Preconditioned Neurobasal/B27 is required to feed primary cultured neurons.

Rat hippocampal neurons were prepared from Sprague-Dawley embryonic rats (E17 to E21) in accordance with the guidelines of the Max Planck Institute of Biochemistry. Hippocampi were dissected, transferred to ice-cold CMF-HBSS-Hepes, minced, transferred to 0.25% Trypsin and 0.1% (w/v) DNase I in CMF-HBSS, and incubated for digestion for 15 min at 37°C. To wash out enzymes, CMF-HBSS medium was replaced three times and tissue was triturated. To remove left over clumps of biological material, the cell solution was passed through a 70-μm cell strainer (Becton Dickinson and Company). After centrifugation (120*g* for 10 min), cells were resuspended in neuronal plating medium (minimal essential medium with Earl’s salt and l-glutamine, 0.6% d-glucose, and 5% fetal bovine serum; Invitrogen) and seeded to a concentration of 3.0 × 10^5^ cells per well on EM grids and glass bottom dishes (MatTek Corp.) for immunohistochemistry. After 4 hours of settling cells on the substrate in a CO_2_ incubator, preconditioned Neurobasal/B-27 medium was added. Three days after cell seeding, 5 μM AraC in preconditioned Neurobasal/B27 was added to each culture dish. One-third of medium was exchanged once a week with Neurobasal/B27 and preconditioned Neurobasal/B27 in equal parts. At DIV 21, just before vitrification, cultures were treated by 1 μM PDBu (final concentration) for 2 min.

### Vitrification of synaptosomes from Munc13- and SNAP25-deficient slice cultures and RIM conditional knockout cultures

Holey gold EM grids (Quantifoil R 2/1, 200 mesh; Quantifoil) were plasma cleaned in a Turbo Sputter Coater Med 010 (Balzers) for 2 min. To make fiducial marker solution, BSA-coated 10-nm gold nanoparticle solution (Aurion 210.133) was concentrated by two centrifugations at 16,000*g* for 25 min (at 4°C), and the pellet was resuspended first in Ca^2+^-free HBM and the second time in HBM + 1.2 mM CaCl_2_. Warm fiducial marker solution was mixed with synaptosomes at 1:10 ratio. Four microliters of the mixture was deposited on each grid, allowed settle down for approximately 7 s, blotted at a small angle for 5 to 7 s with a filter paper (Whatman filter paper 1, qualitative circles, 90 mm in diameter, catalog no. 1001 090) and vitrified by plunge freezing into liquid nitrogen (LN_2_)–cooled pure ethane (Westfalen AG, ethane 2.5 99.5% vol % C_2_H_6_) using a portable manual plunger (designed and built by Max Planck’s workshop). Vitrified grids were stored in LN_2_ until EM imaging.

### Vitrification of neocortical synaptosomes

Holey copper or molybdenum EM grids (Quantifoil R 2/1, 200 mesh) were glow-discharged (Harrick Plasma cleaner PDC-3XG) for 45 s. To make fiducial marker solution, BSA-coated 10-nm Au nanoparticle solution (Aurion, 210.133) was concentrated four times by two centrifugations at 14,000*g* for 60 min, and the pellet was resuspended in Ca^2+^-free HBM. Warm fiducial marker solution was mixed with synaptosomes at 1:10 ratio. Four microliters of the mixture was deposited on each grid and vitrified by plunge-freezing into a liquid ethane/propane mixture using Vitrobot Mark III or Vitrobot Mark IV (Thermo Fisher Scientific). The vitrobot settings were as follows: blot offset, −3 (Mark III); blot force, 10 (Mark IV); blotting time, 10 s; at 37°C; and 95% humidity. Vitrified grids were stored in LN_2_ until EM imaging.

### Vitrification of WT neuronal cultures

Primary cultured neurons were seeded onto holey gold EM grids (Quantifoil R 1/4 gold, 200 mesh). The fiducial marker solution was prepared as for neocortical synaptosomes. Four microliters of the solution was applied to grids on which neurons were grown. They were vitrified using Vitrobot Mark IV (Thermo Fisher Scientific) using the same settings as for neocortical synaptosomes, except that a waiting time of 5 s was imposed to allow fiducial markers to diffuse on the sample. Vitrified grids were stored in LN_2_ until EM imaging.

### Cryo–focused ion beam

Vitrified WT neuronal cultures grown on EM grids were thinned by FIB using Quanta 3D field emission gun and Scios DualBeam, FEI dual-beam microscopes, equipped with an 360° rotatable cryo-stage operated at −180°C, as described before (*108*). Grids were mounted in FEI Autogrids (FEI), modified for shallow milling angles (*52*), and placed in a cryo-FIB shuttle (*109*). The additional carbon layer evaporated on the grids before plating neuronal cultures helped finding the correct orientation. The cryo-FIB shuttle was transferred to the microscope using a cryo-transfer system (PP3000Q, Quorum). For initial experiments grids were sputtered (10 mA for 60 s) with a platinum layer to prevent surface charging of specimen. The effect of uneven milling (curtaining) was avoided by applying a protective layer of platinum onto the grid by a gas injection system. The region of interest was monitored by the electron beam at 5 keV and milled with 30-keV gallium ions. In general, rough milling (0.3 to 0.5 nA) was used to create rectangular holes surrounding the region of interest, and the milled area was further thinned (0.1 nA). Currents between 30 and 50 pA were used to polish milled surfaces. Alternatively, the cleaning cross-sectional milling strategy was performed at 0.1 nA, where a selected area is milled line by line, using *z* size of 6 μm, 700-ns dwell time, and 65% overlap.

Both the wedge and lamella FIB milling strategies were used. Wedges were milled in a relatively wide pattern of 35 μm, typically at 5° milling angle. Because of their shape, wedges contain only a small region close to the edge that is sufficiently thin for cryo-ET (fig. S7A). In addition, the extended culturing time needed to ensure synaptogenesis often results in neurons growing processes on the bottom side of the EM grid carbon support (neurons are plated on the top side), thus making an additional thickness that cannot be removed by cryo-FIB milling because that would require unfeasibly low milling angles.

Lamellas, being more fragile than wedges, were milled to the width of 15 μm. However, extended regions or even the entire length of a lamella can be milled to a suitable thickness. The milling was typically performed at 11°, and the desired lamella thickness just before the polishing step was 1 μm. Because lamellae are more fragile than wedges, densely grown cultures are required for a sufficient support, but the increased sample thickness often caused unsatisfactory vitrification.

Furthermore, cryo-FIB thinning of neuronal processes, especially at low milling angles as required for wedges, resulted in material redeposition and surface contamination. Although we removed the surface contamination-induced 3D reconstruction artifacts using a previously developed software (*54*), the contamination obscured the view to the interior of thinned regions, aggravating synapse detection.

### Cryo-ET acquisition and reconstruction

Tilt series were collected under a low-dose acquisition scheme using SerialEM (*110*, *111*) on Titan Krios and Polara microscopes (Thermo Fisher Scientific) equipped with a field emission gun operated at 300 kV, with a postcolumn energy filter (Gatan) operated in the zero-loss mode (20-eV slit width) and with a computerized cryostage designed to maintain the specimen temperature of <−150°C. Tilt series were typically recorded from −60° to 60° with a 1.5° to 2° angular increment, using a modified version of the dose-symmetric scheme (*112*) or in two halves starting from 0°, and the total dose was kept <100 *e*^−^/Å^2^. Almost all tilt series were recorded on a direct electron detector device (K2 Summit, Gatan) operated in the counting mode. Pixel sizes was 0.34 and 0.44 nm at the specimen level. Volta phase plate with nominal defocus of 0.5 to 1 μm (*113*) was used. Individual frames were aligned using Motioncor2 (*114*). A few tilt series of neocortical synaptosomes were recorded on a 2000 × 2000 charge-coupled device camera (MegaScan, Gatan) at 5-μm nominal defocus. Tilt series were aligned using gold beads as fiducial markers, and 3D reconstructions were obtained by weighted back projection using Imod (*115*). During reconstruction, the projections were binned using a factor of 4 (final pixel size, 1.368 to 1.756 nm) and low pass–filtered at the postbinning Nyquist frequency.

Cryo-FIB milling of neuronal cultures caused a strong redeposition of milled material. To remove 3D reconstruction artifacts induced by the redeposition, we applied the software procedure previously developed for this purpose (*54*). The number of iterations was set to five.

### Selection of tomograms

We selected for further processing tomograms that were of sufficient technical and biological quality. Specifically, tomograms were deemed technically acceptable if they did not contain any signs of ice crystal formation such as ice reflections or faceted membranes, and they had reasonable signal-to-noise ratio and proper tomographic alignment. We discarded synapses showing signs of deterioration such as elliptical small vesicles or strong endocytotic features. Only synaptosomes containing a presynaptic mitochondrion showing intact outer and inner membranes and cristae were kept. The selected synapses contained uninterrupted and smooth presynaptic and postsynaptic membranes, and the pre- and postsynaptic terminals were separated by a cleft of approximately 25 nm in width, containing trans-cleft protein complexes (*23*).

### Detection, segmentation, and analysis of tethers, connectors, and SVs

SV-associated complexes, tethers and connectors, were detected and localized in an automated fashion and analyzed using Pyto package as described before (*21*, *45*). Briefly, the AZ membrane was manually segmented in Amira (Visualization Sciences Group). SVs were segmented by manually tracing the maximum diameter profile and automatically extending it to a sphere. Hierarchical connectivity segmentation finds connected clusters of pixels linking two different membranes in 3D at multiple thresholds, followed by selecting clusters at the lowest threshold, thus providing a valid segmentation. In this way, the segmented tethers (segments linking SVs to plasma membrane) and connectors (segments interlinking SVs) represent the “core” structure of biological tethers and connectors.

For the analysis of vesicle distribution, the presynaptic cytoplasm (including SVs) was divided into 1-pixel-thick layers according to the distance to the AZ membrane, and the fraction of the layer volume occupied by SVs was measured. The surface concentration of SVs was calculated as the number of SVs in the proximal zone divided by the surface area of the AZ membrane. The SV distance to the AZ membrane was calculated as the shortest distance between the AZ membrane and SV pixels.

Analysis of detected tethers and connectors included the determination of their morphology, precise localization, and their interrelationship. Connector and tether lengths were calculated as the minimal edge-to-edge distance between connector and tether voxels contacting an SV or plasma membrane that takes into account central regions of tethers and connectors, as previously described (*45*). In this way, the ambiguity inherent to the measurement of length of 3D objects is resolved and curvature of tethers and connectors contributed to their calculated lengths. However, possible extended protein-lipid binding regions are not considered, making it possible that different lipid binding modes influence tether length determination. As expected, tether length was larger than the proximal SV distance to the AZ zone for all conditions (fig. S1, B to E) because many tethers were curved or they connected a side of an SV with the AZ membrane (Fig. 3C). All features were analyzed within 250 nm from the AZ membrane.

To account for the missing wedge, we determined the synapse orientation as the direction of the vector perpendicular to the pre- and postsynaptic membranes in respect to the *x* axis (these vectors were in *x*-*y* plane for all synapses). Although there were no significant differences between the experimental conditions, we removed as many synapses as needed to equalize the mean angles between the conditions (synapses were removed in the order from the most extreme angle). This resulted in the removal of three SNAP25 KO, two Munc13 DKO + PDBu, two plain (−Ca^2+^), and two Ro31-8220 synapses from all analysis steps that involved tethers and connectors.

All image processing and statistical analysis software procedures were written in Python and implemented in Pyto package (*45*). Pyto uses NumPy and SciPy packages and graphs are plotted using Matplotlib (*116*–*118*).

### Fitting atomic models

Tethers were extracted using Pyto package in two forms, directly from tomograms as grayscale densities (grayscale tethers) and as binary segments obtained by our hierarchical connectivity procedure (segmented tethers) (*45*). Tethers were segmented in a fully automated manner; hence, their isosurface levels were not manually adjusted for fitting.

Two atomic models of the SNARE complex were used for fitting, both comprising one SNARE motif each of VAMP2 and Syntaxin 1a, two SNARE motifs of SNAP25, a helical fragment of Complexin 1, and the C_2_B domain of Synaptotagmin 1. In the first model, the C_2_B domain is located at the tripartite interface [PDB ID: 5w5d (*62*)] (Fig. 4A). The second model contains the C_2_B domain at the primary interface. To obtain this model, we superposed PDB IDs: 1KIL (*60*) and 5CCH (*61*), so that the SNARE complex helices of the two models were aligned and kept Complexin 1 (1KIL) and the primary interface C_2_B domain (5CCH). To fit Munc13, we used the fragment comprising C_1_, C_2_B, and MUN domains that covers 55% of the entire Munc13 sequence [PDB ID: 5ue8 (*64*)] (Fig. 4A).

We performed rigid body fitting of the selected atomic models, as follows. First, we performed a rough manual fit of atomic models in the segmented tethers, and then we used the automated fit using UCSF ChimeraX software (*119*). If needed, fits were further adjusted manually. In all cases, we made sure that the global orientation of the atomic models was correct, namely, the C termini of SNARE helices were localized close to the tether contacts with both SV and plasma membranes and that N terminus of the Munc13 C_1_C_2_BMUN model was oriented toward the plasma membrane and the C-terminal toward the SV. In addition, the atomic models were not allowed to overlap with the lipid membranes, examples of unsuccessful fits are shown in Fig. 4C. Last, to avoid false negatives that might arise because tethers are segmented at the lowest grayscale level at which the tether is detected and thus may underestimate the real (grayscale) tether volume (*45*), we determined whether the models could fit the grayscale tether.

In this way, we tested whether tethers were of sufficient size, appropriate shape, and orientation to accommodate the entire atomic models. A fit was considered satisfactory even if a part of the tether is not occupied by the model because it is possible that under native conditions, other proteins bind those comprising the atomic models thus contributing to tethers and because the Munc13 C_1_C_2_BMUN model contains 55% of the entire Munc13. Fitting (correlation) scores for successful automated fits at the resolution of 3.0 nm reported by Chimera were generally between 0.75 and 0.85 (1.0 is the perfect score). We considered a fit unsuccessful if with our best effort, significant parts of the model could not fit into a tether. The determined tether length did not influence the fitting procedure because fitting takes into account entire tethers.

Specifically, we found that in all 25 of 80 cases where segmented short tethers were too small to accommodate SNARE complex helices, grayscale tethers provided an adequate fit. Fitting the Munc13 C1C2BMUN model into intermediate tethers of WT, Munc13 DHet, WT + PDBu, and SNAP25 KO synapses, 30 of 210 grayscale tethers could not accommodate the model (39 of 210 when considering only segmented tethers). Considering only SNAP25 KO synapses, 5 of 41 grayscale tethers could not accommodate the model (6 of 41 when considering only segmented tethers). Furthermore, grayscale tethers could not accommodate the mode in 5 of 41 cases (6 of 41 segmented tethers) in SNAP25 KO and in 16 of 31 (17 of 31 segmented tethers) in intact neurons. Because the fairly large N-terminal region of Munc13 (540 amino acids) is not included in the Munc13 C_1_C_2_BMUN model, its conformation with respect to the C_1_C_2_BMUN region is not known; hence, a successful fitting of the Munc13 C_1_C_2_BMUN model into a tether constitutes only a necessary condition for the tether to contain Munc13. Therefore, the number of tethers that cannot accommodate Munc13 may be higher than what we determined.

### Statistical analysis

Statistical analysis was performed between the experimental groups using only planned, orthogonal comparisons. Specifically, Munc13 DKO was compared to Munc13 DHet, SNAP25 KO to SNAP25 WT, WT + PDBu to WT, Munc13 DKO + PDBu to Munc13 DKO, SNAP25 KO + PDBu to SNAP25 KO, and all three −Ca^2+^ treatments (PDBu, calphostin C + PDBu, and Ro31-8220 + PDBu) to plain (−Ca^2+^). The numbers of synapses, vesicles, connectors, and tethers analyzed for each category are shown in table S1. The intended number of synapses per experimental group was 5 to 10. The actual numbers varied because of the combination of the factors: (i) Tomograms were acquired in batches of the same condition, (ii) tomogram reconstruction and selection did not immediately follow the acquisition, and (iii) all tomograms that satisfied the selection criteria were included in the analysis. For the analysis of properties pertaining to individual SVs, connectors and tethers (such as the SV distance to the AZ membrane, tether length, and fraction of tethers/connectors having a certain property), values within experimental groups were combined. Bars on the graphs show mean values, and error bars show the SEM. In cases, a fraction of SVs or tethers is shown, and the error bars represent SEM between synapse means. We used Student’s *t* test for statistical analysis of values that appeared to be normally distributed (e.g., vesicle diameter) and Kruskal-Wallis test (nonparametric) for values deviating from the normal distribution (e.g., number of tethers and connectors per vesicle). For frequency data (e.g., fraction of connected and nonconnected vesicles), χ^2^ test was used. In all cases, confidence levels were calculated using two-tailed tests. The confidence values were indicated in the graphs by *P < 0.05, **P < 0.01, and ***P < 0.001.

## Notes

View/request a protocol for this paper from *Bio-protocol*.
