# Supplementary material for: Munc13- and SNAP25-dependent molecular bridges play a key role in synaptic vesicle priming
Source: Sci Adv. 2023 Jun 21;9(25):eadf6222. doi: 10.1126/sciadv.adf6222 (PMC10284560; doi:10.1126/sciadv.adf6222)
Supplement: Supplementary file 1 — Table S1 Figs. S1 to S7 Legends for movies S1 to S3 [file sciadv.adf6222_sm.pdf]

Science Advances
AAAS

Supplementary Materials for

Munc13- and SNAP25-dependent molecular bridges play a key role in synaptic vesicle priming

Christos Papantoniou *et al.*

Corresponding author: Vladan Lučić, vladan@biochem.mpg.de

Sci. Adv. **9**, eadf6222 (2023)
DOI: 10.1126/sciadv.adf6222

The PDF file includes:

Table S1
Figs. S1 to S7
Legends for movies S1 to S3

Other Supplementary Material for this manuscript includes the following:

Movies S1 to S3

	N synapses	N proximal SVs	N tethers	N proximal connectors
Munc13 and SNAP25				
WT	14	182	555	630
WT +PDBu	8	59	200	102
Munc13 DHet	8	80	266	253
Munc13 DKO	13	96	117	356
Munc13 DKO +PDBu	9 / 7	62	61	184
SNAP25 WT	9	110	349	340
SNAP25 KO	14 / 11	147	300	471
SNAP25 KO +PDBu	7	124	457	625
Pharmacologically treated				
Plain (-Ca^{2+})	17 / 15	228	254	244
PDBu (-Ca^{2+})	16	164	270	164
Calphostin C + PDBu (-Ca^{2+})	9	87	107	101
Ro31-8220 + PDBu (-Ca^{2+})	8 / 6	118	167	121
RIM				
RIM Ctrl	4	34	188	
RIM cDKO	5	10	39	
Neuronal cultures				
	5	39	108	

Table S1: Number of analyzed proximal SVs, tethers and proximal connectors. In cases where synapses were removed to equalize the mean angular orientation, the number of synapses before / after the removal are specified. The numbers of tethers and connectors always include only the retained synapses. "Munc13 and SNAP25" denote organotypic slice culture synaptosomes, "Pharmacologically treated" denote neocortical synaptosomes, "RIM" denote dissociated culture synaptosomes and "Neuronal culture" denote synapses from intact neurons.

Figure S1

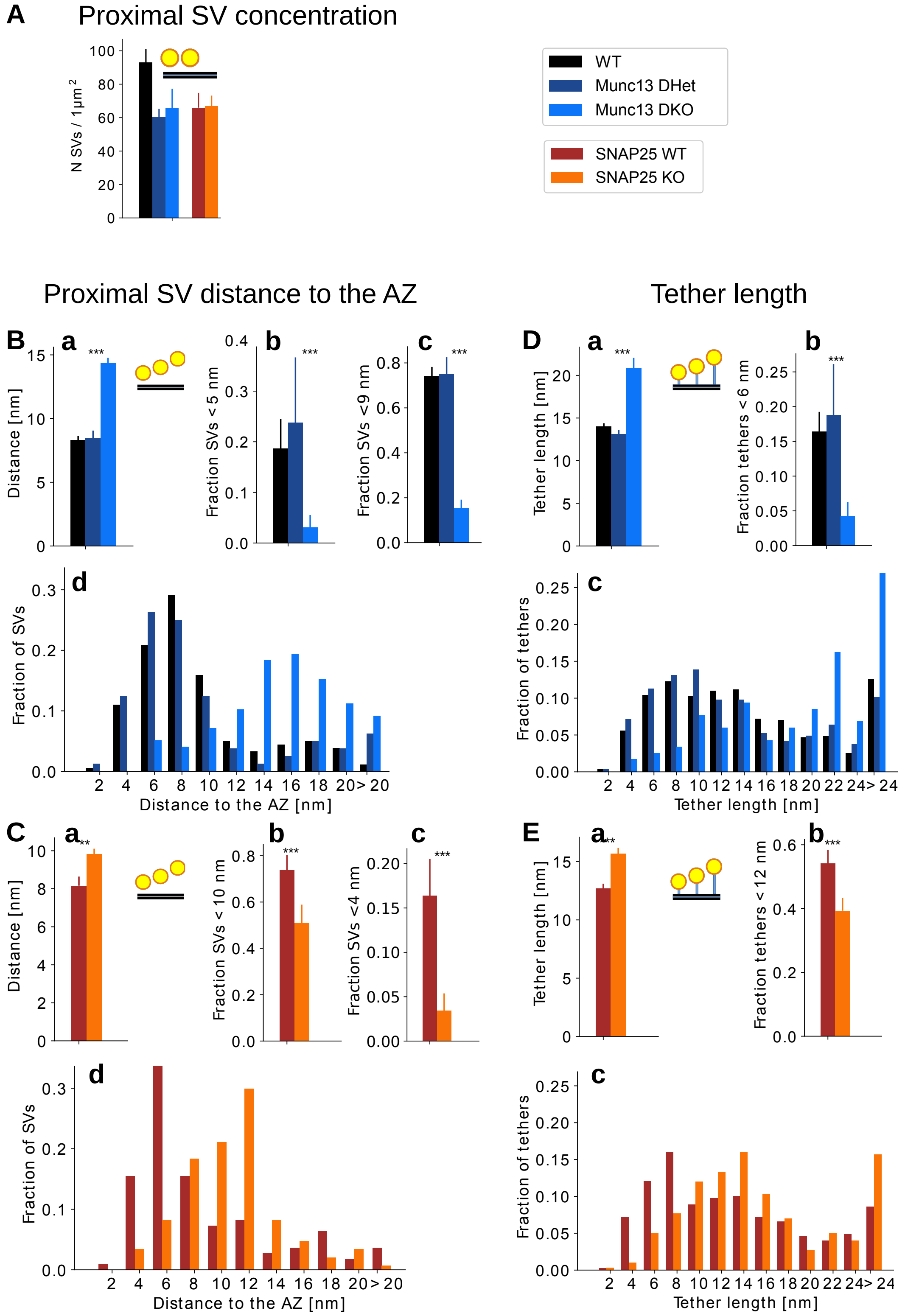
A
Proximal SV concentration
N SVs / 1μm²
WT
Munc13 DHet
Munc13 DKO
SNAP25 WT
SNAP25 KO
Proximal SV distance to the AZ
Tether length
B
a
Distance [nm]
***
b
Fraction SVs < 5 nm
c
Fraction SVs <9 nm
D
Tether length [nm]
Fraction tethers < 6 nm
d
Fraction of SVs
Distance to the AZ [nm]
Fraction of tethers
Tether length [nm]
C
Fraction SVs < 10 nm
Fraction SVs <4 nm
E
Fraction tethers < 12 nm

Figure S1: Influence of Munc13 and SNAP25 on the proximal SV location and tether length, additional data. (A) The surface concentration of the proximal SVs at the AZ. (B) Proximal SV distance to the AZ membrane (a), fraction of proximal SVs located <5 nm to the AZ membrane (b), <9 nm to the AZ membrane (c) and the histogram of the SV distances (d), all from Munc13-related conditions. (C) Proximal SV distance to the AZ membrane (a), fraction of proximal SVs located <10 nm to the AZ membrane (b), <4 nm to the AZ membrane (c) and the histogram of the SV distances (d), all from SNAP25-related conditions. (D) Tether length (a), fraction of tethers shorter than 6 nm (b) and histogram of tether lengths (c), all from Munc13 conditions. (E) Tether length (a), fraction of tethers shorter than 12 nm (b) and histogram of tether lengths (c), all from SNAP25-related conditions.

Figure S2

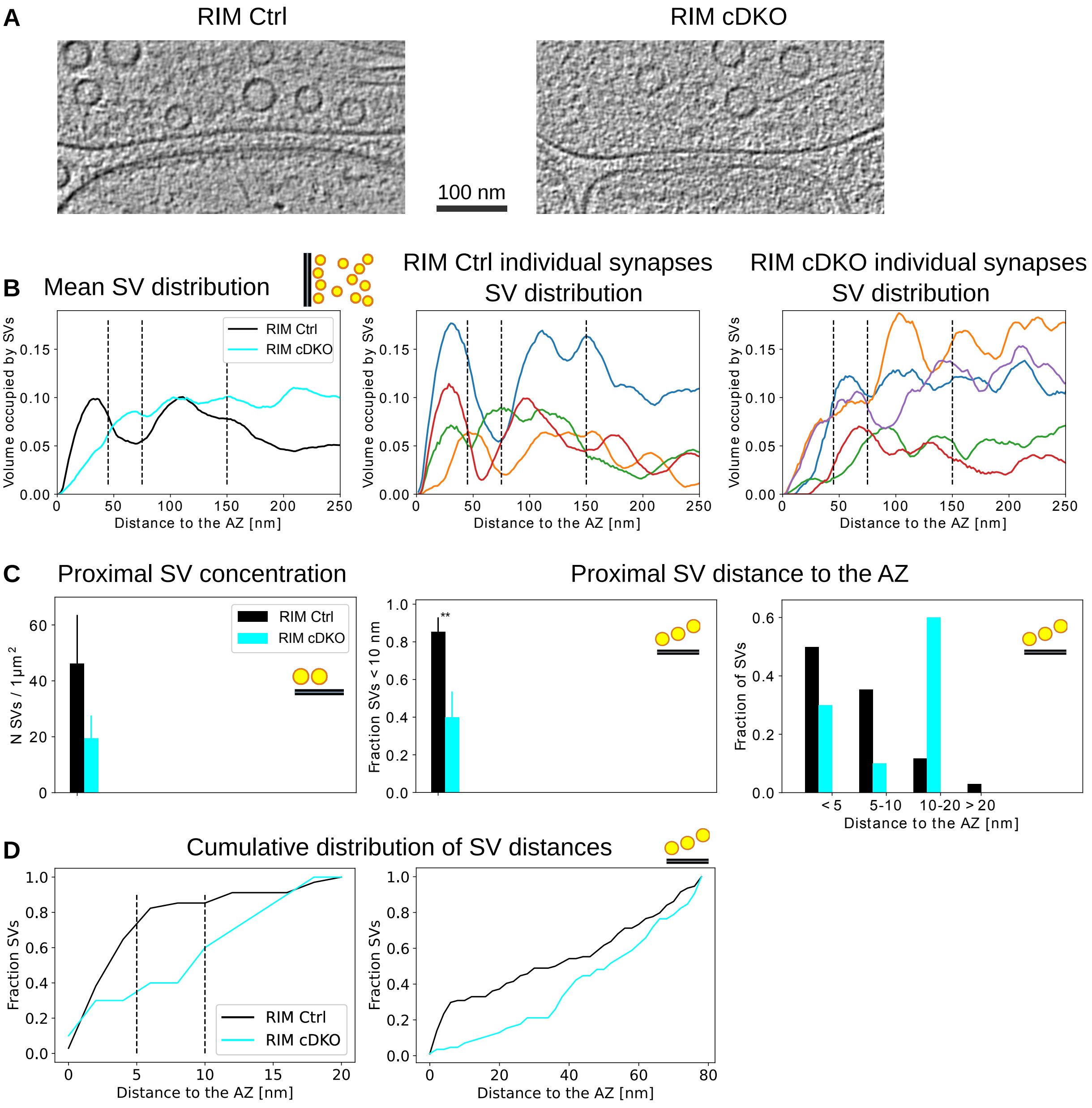

Figure S2: Role of the RIM family for SV localization. (A) Cryo-ET slices of RIM Ctrl and RIM cDKO synapses. (B) SV distribution up to 250 nm to the AZ membrane, mean values (left). Distributions from individual RIM Ctrl (N=4, middle) and RIM cDKO (N=5, right) synapses, the actual colors are irrelevant as they only distinguish individual synapses (blue, orange, green, red, violet). Dashed black lines show separation between proximal intermediate and distal SVs. (C) Proximal SVs, the surface concentration at the AZ (left), fraction of SVs <10 nm to the AZ membrane (middle) and the histogram of the distances (right). (D) Cumulative distribution function of the SV distances up to 20 nm (left) and 80 nm (right). Black, dashed lines show distance regions that characterize proximal SVs of WT, SNAP25 KO and Munc13 DKO synapses.

Figure S3

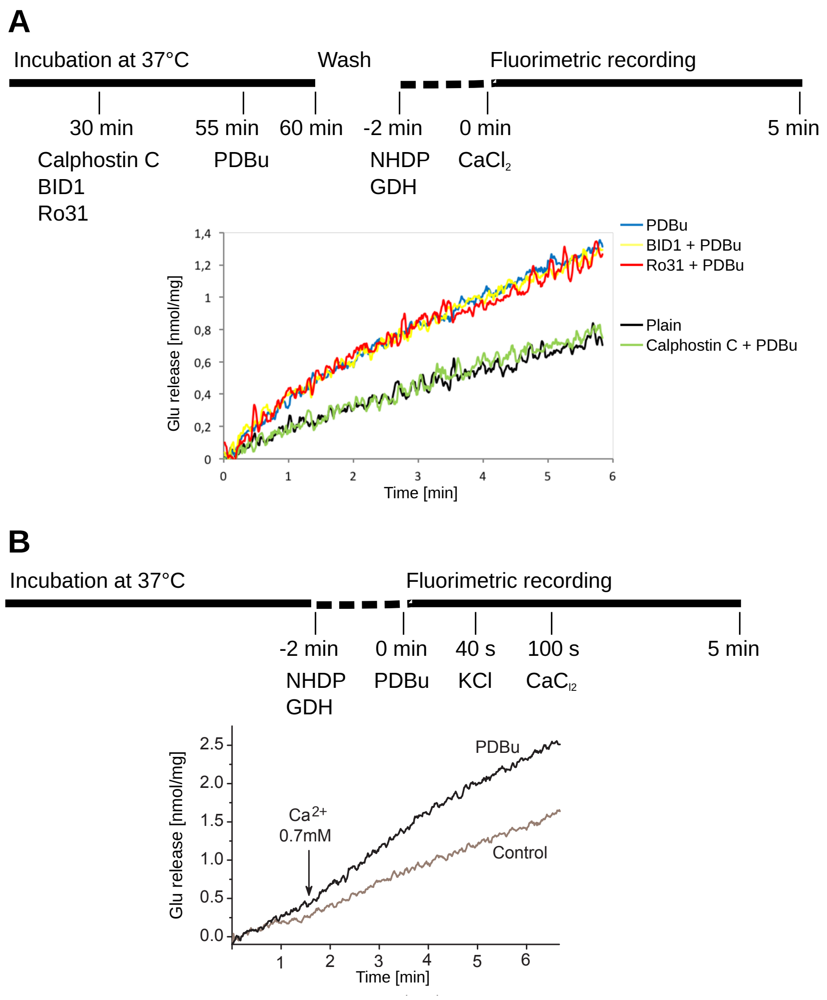

Figure S3: Glutamate release in neocortical synaptosomes. (A) Spontaneous release in pharmacologically treated synaptosomes (number of experiments 5-7 per treatment). (B) The influence of extracellular Ca^{2+} under a mild stimulation (5 mM KCl) and in the presence of PDBu. The exact timings are shown above the graphs.

Figure S4

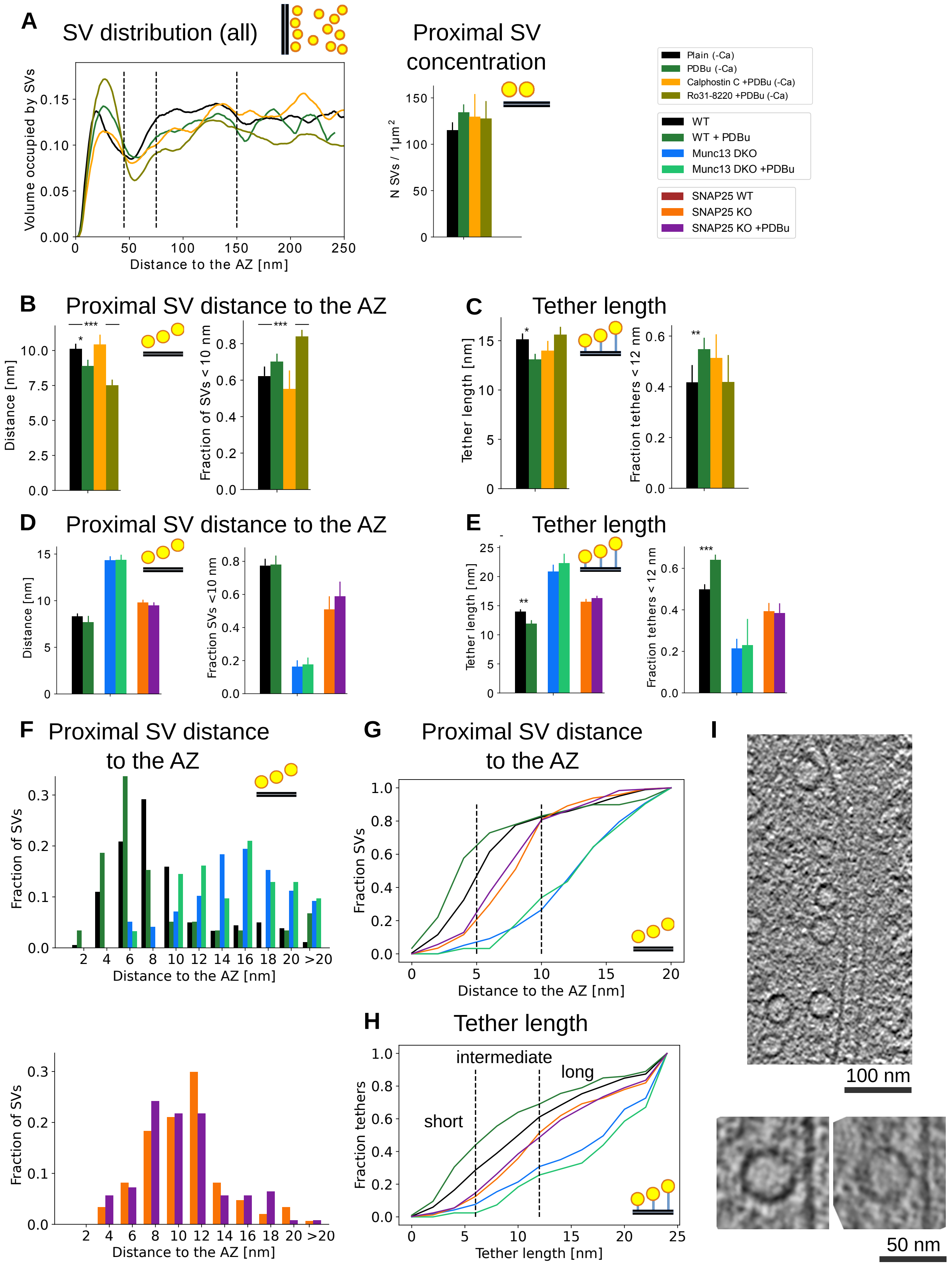
A
SV distribution (all)
Volume occupied by SVs
Distance to the AZ [nm]
Proximal SV concentration
N SVs / 1μm²
Plain (-Ca)
PDBu (-Ca)
Calphostin C +PDBu (-Ca)
Ro31-8220 +PDBu (-Ca)
WT
WT + PDBu
Munc13 DKO
Munc13 DKO +PDBu
SNAP25 WT
SNAP25 KO
SNAP25 KO +PDBu
B
Proximal SV distance to the AZ
Distance [nm]
Fraction of SVs < 10 nm
C
Tether length
Tether length [nm]
Fraction tethers < 12 nm
D
Proximal SV distance to the AZ
Distance [nm]
Fraction SVs <10 nm
E
Tether length
Tether length [nm]
Fraction tethers < 12 nm
F
Proximal SV distance to the AZ
Fraction of SVs
Distance to the AZ [nm]
G
Proximal SV distance to the AZ
Fraction SVs
Distance to the AZ [nm]
H
Tether length
intermediate
long
short
Fraction tethers
Tether length [nm]
I
100 nm
50 nm

Figure S4: Influence of PDBu on SV distribution and tether length. (A) Mean SV distribution up to 250 nm to the AZ membrane (left) and the surface concentration of the proximal SVs at the AZ (right). (B) Proximal SV distance to the AZ membrane (left) and fraction of proximal SVs located $<$10 nm to the AZ membrane (right). (C) Tether length (left) and the fraction of tethers shorter than 12 nm (right). (D) Proximal SV distance to the AZ membrane (left) and fraction of proximal SVs located $<$5 nm to the AZ membrane (right). (E) Tether length (left) and the fraction of tethers shorter than 12 nm (right). (A-C) pharmacologically treated neocortical synaptosomes, (D-E) Munc13 and SNAP25 conditions. (F) Histograms of SV distances. (G) Cumulative distribution function of the SV distances, where the distances used to classify the proximal SV distances are shown by black, dashed lines. (H) Cumulative distribution function of tether lengths, where the lengths used to classify tethers are shown by black, dashed lines. (I) Cryo-ET slices from WT +PDBu synapses.

Figure S5

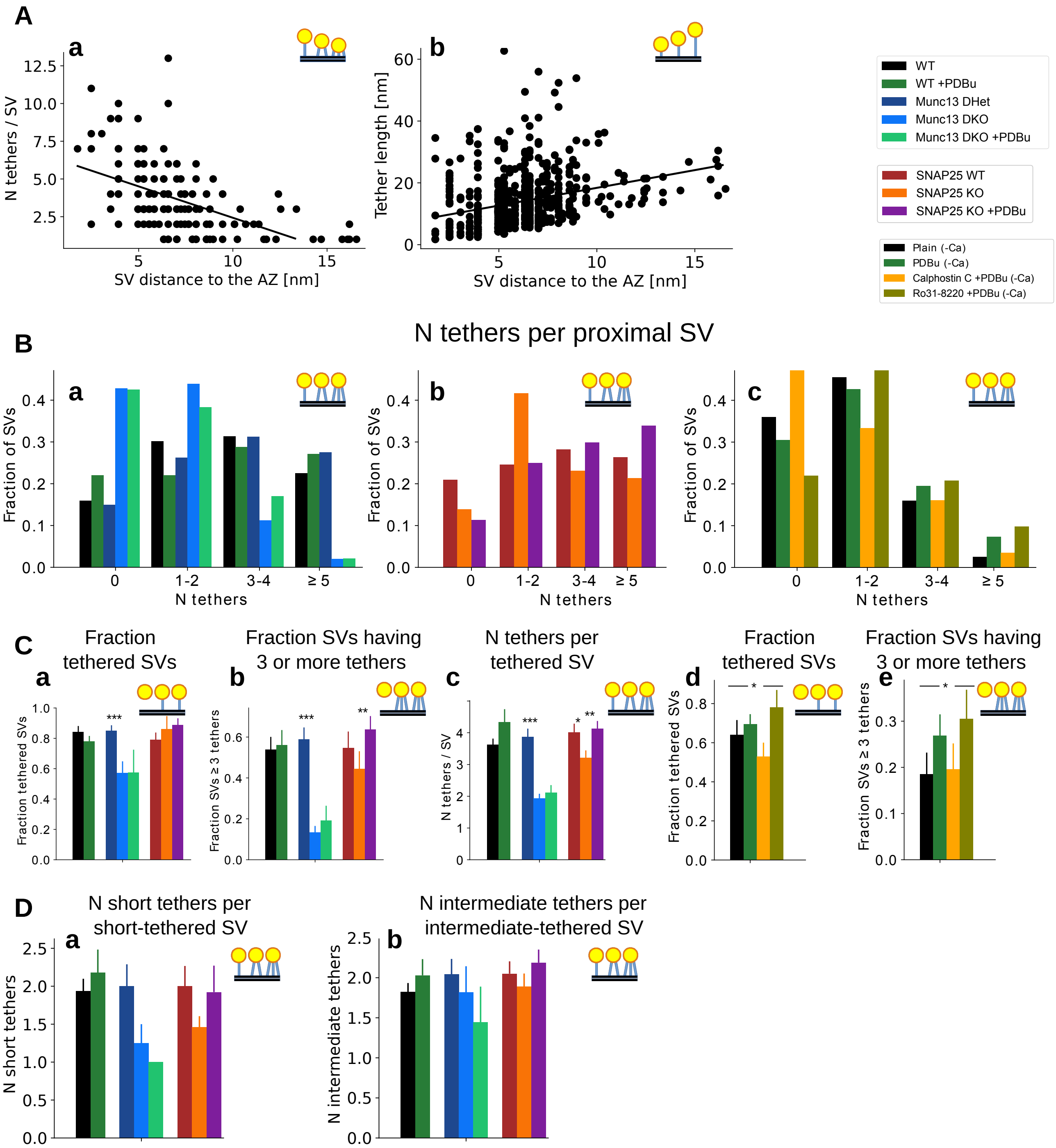

Figure S5: SV tethering. (A) Correlations between the number of tethers per proximal SV and the SV distance to the AZ (a), and the tether length and the SV distance to the AZ (b) in WT. (B) Histograms of the number of tethers per proximal SV. (C, D) Characterization of tethering, as indicated on the graphs.

Figure S6

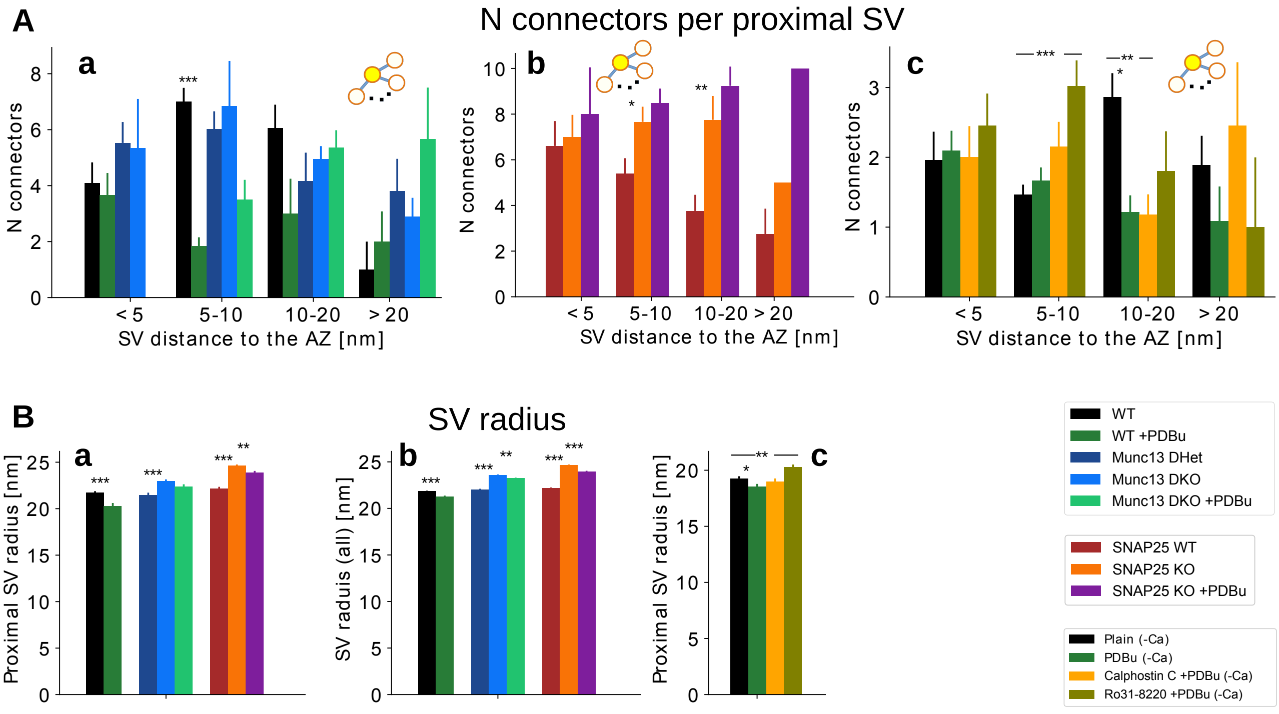

Figure S6: SV connectivity and size. (A) The number of connectors per proximal SV for different SV classes. (B) SV radius for proximal SVs (a and c) and for all SVs within 250 nm to the AZ membranes (b). In all cases, the data for Munc13 and SNAP25 conditions are shown on the left and for the pharmacologically treated neocortical synaptosomes on the right.

Figure S7

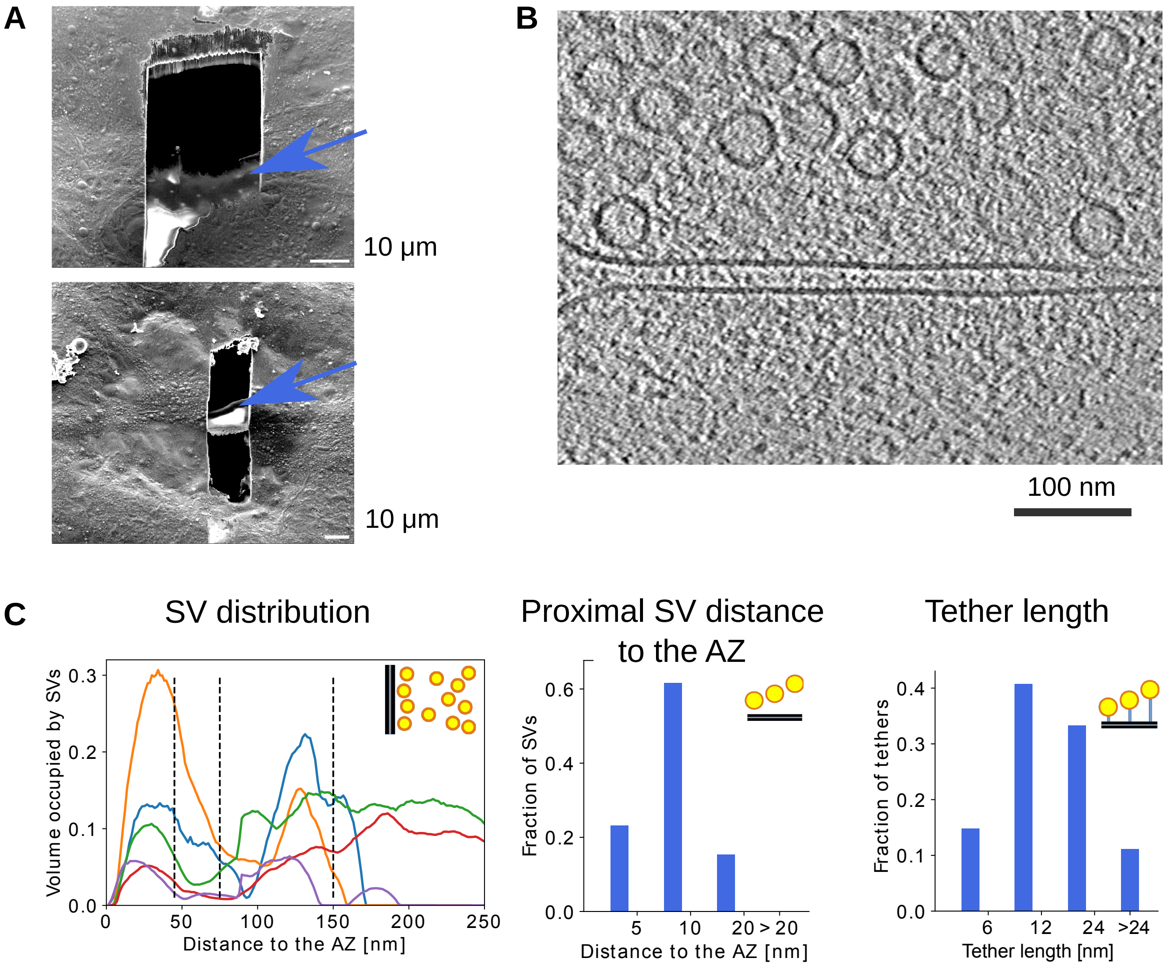

Figure S7: Intact neurons. (A) SEM images of a cryo-FIB milled wedge (above) and lamella (below). Arrows indicate the areas that are sufficiently thin for cryo-ET imaging. (B) Cryo-ET slice of a synapse. (C) SV distribution up to 250 nm to the AZ membrane of individual synapses, where all synapses were treated by PDBu and colors indicate different synapses (left). Histograms of the SV distances to the AZ membrane (center) and tether lengths (right).

Supplementary video 1: Cryo-ET image of a WT synapse, followed by 3D rendering of SVs (blue), the AZ membrane (blue), segmented tethers (violet) and connectors (green).

Supplementary video 2: The same as Supplementary video 1, except that a Munc13 DKO synapse is shown.

Supplementary video 3: The same as Supplementary video 1, except that a synapse from intact neuronal cultures is shown.
